# Gut microbiota affects the estrus return of sows by regulating the metabolism of sex steroid hormones

**DOI:** 10.1186/s40104-023-00959-5

**Published:** 2023-12-20

**Authors:** Min Liu, Jia Zhang, Yunyan Zhou, Shuqi Xiong, Mengqing Zhou, Lin Wu, Qin Liu, Zhe Chen, Hui Jiang, Jiawen Yang, Yuxin Liu, Yaxiang Wang, Congying Chen, Lusheng Huang

**Affiliations:** National Key Laboratory of Swine Genetic Improvement and Germplasm Innovation, Jiangxi Agricultural University, Nanchang, 330045 China

**Keywords:** Estrus return, Fecal metabolome, Gut microbiota, Metagenomics, Sex steroid hormones, Sow

## Abstract

**Background:**

Sex hormones play important roles in the estrus return of post-weaning sows. Previous studies have demonstrated a complex and bi-directional regulation between sex hormones and gut microbiota. However, the extent to which the gut microbiota affects estrus return of post-weaning sows is largely unknown.

**Results:**

In this study, we first screened 207 fecal samples from well-phenotyped sows by 16S rRNA gene sequencing and identified significant associations between microbes and estrus return of post-weaning sows. Using metagenomic sequencing data from 85 fecal samples, we identified 37 bacterial species that were significantly associated with estrus return. Normally returning sows were characterized by increased abundances of *L. reuteri* and *P. copri* and decreased abundances of *B. fragilis*, *S. suis*, and *B. pseudolongum*. The changes in gut microbial composition significantly altered the functional capacity of steroid hormone biosynthesis in the gut microbiome. The results were confirmed in a validation cohort. Significant changes in sex steroid hormones and related compounds were found between normal and non-return sows via metabolome analysis. An integrated analysis of differential bacterial species, metagenome, and fecal metabolome provided evidence that normal return-associated bacterial species *L. reuteri* and *Prevotella* spp. participated in the degradation of pregnenolone, progesterone, and testosterone, thereby promoting estrogen biosynthesis. Furthermore, the microbial metabolites related to sow energy and nutrient supply or metabolic disorders also showed relationships with sow estrus return.

**Conclusions:**

An integrated analysis of differentially abundant bacterial species, metagenome, and fecal metabolome revealed the involvement of *L. reuteri* and *Prevotella* spp. in sow estrus return. These findings provide deep insight into the role of gut microbiota in the estrus return of post-weaning sows and the complex cross-talk between gut microbiota and sex hormones, suggesting that the manipulation of the gut microbiota could be an effective strategy to improve sow estrus return after weaning.

**Supplementary Information:**

The online version contains supplementary material available at 10.1186/s40104-023-00959-5.

## Background

The period from the time that nursing piglets are removed from the sow to the time that the sow shows the first estrus behaviors is defined as the weaning-to-estrus interval. An excessively long weaning-to-estrus interval increases the number of non-productive days (NPDs, referring to the days that the sow is not pregnant or lactating), reduces the number of weaned piglets per sow per year (PSYs), and results in significant economic losses. The weaning-to-estrus interval is an important indicator reflecting the normal return of estrus after weaning. About 85% of sows will display estrus within 7 d after weaning [1]. However, certain sows fail to return to estrus for a long period after weaning, a phenomenon that remains to be addressed in the pig industry.

The hypothalamic-pituitary-ovarian (HPO) axis feedback loop plays a crucial role in the regulation of the endocrine system and sow estrus. The composition, functional capacity, and metabolites involved have been associated with a multitude of physiological systems, including the endocrine and reproductive systems [2]. Previous studies have investigated the influence of gut microbiota on the regulation of sex steroid hormones [3], the duration of farrowing, and the post-weaning estrus interval in sows [4]. A complex interplay exists between gut microbiota and sex steroid hormones. Sex steroid hormones shape the gut microbiota by regulating the innate immune response [5, 6]. Conversely, the gut microbiota modulates local and systemic levels of sex steroid hormones by synthesizing enzymes that can modify these molecules [7]. The gut microbiota is a major regulator of androgen metabolism [8]. The enzyme 3β-hydroxysteroid dehydrogenase expressed by gut microbes degrades testosterone and has been linked to depression in males [9]. Bacterial-derived β-glucuronidase, β-glucosidase, and hydroxysteroid dehydrogenases (HSD) can convert the conjugated estrogen into a free form that promotes the reabsorption of estrogen into the enterohepatic circulation where it exerts multiple biological functions [10]. The dysbiosis of the gut microbiota is involved in a wide range of diseases in women, including bacterial vaginosis, infertility, gestational diabetes, polycystic ovary syndrome (PCOS), and obesity [11]. Studies in mice have demonstrated significant effects of gut microbiota on the host estrus cycle. For example, depletion of the gut microbiota by antibiotics resulted in an altered estrus cycle in female mice [12]. Compared with the conventionally raised mice with a natural gut microbiota, germ-free mice showed abnormal estrus cycles [13]. However, there are few related studies in pigs.

Elucidating the underlying mechanisms of the intestinal microbiota and their metabolites influencing the estrus return of sows after weaning has significance for the pig industry. Our previous study suggested that the gut microbiota was associated with the shifts of serum metabolites and that this may influence the interval from weaning to estrus [14]. With the development of high-throughput multi-omics technologies, shotgun metagenomic sequencing can provide a profile of the microbiota composition and functional capacities of the microbiome. Fecal metabolome analysis is a reliable method with which to understand the metabolic activities of the gut microbiota and is an effective complement to functional readout of the gut microbiome. Recently, the multi-omics approach has been applied to explore the effects of gut microbiota on host physiology and diseases such as PCOS [2], major depressive disorders [15], and insulin resistance [16].

In the present study, we investigated the relationship of the gut microbiota with the failure of estrus return after weaning in more than 200 experimental sows, and we examined the underlying mechanisms of the changes in gut microbiota and their metabolites influencing the failure of estrus return by integrating 16S rRNA gene sequencing, shotgun metagenomic sequencing, and fecal metabolome analysis using the study workflow shown in Fig. S1. Based on these analyses, we identified the specific bacterial species and metabolites associated with sow estrus return. We suggest that the dysbiosis of gut microbiota may disturb the level of sow steroid hormones and sex hormone-related compounds and the metabolites related to sow energy and nutrient supply or metabolic disorders, ultimately leading to the failure of estrus return in sows. We also provide biomarkers that could be used to predict non-return sows.

## Materials and methods

### Animals and sample collection

A total of 236 Landrace × Yorkshire sows, most of which were distributed from the fourth to seventh parity, were used in this study. These experimental sows were derived from two independent sow cohorts that were defined as the discovery cohort (*n* = 207) and the validation cohort (*n* = 29). The experimental sows were housed and managed under similar farm conditions and were provided with commercial formula diets containing 67% corn and 26% soybean meal (Table S1). Clean water was supplied ad libitum. All subjects were healthy and did not receive any antibiotics or probiotics for at least 2 months prior to sample collection. A total of 236 fecal samples were collected from the two independent sow cohorts on the day of weaning. Full details concerning fecal sample collection were described previously [14]. Briefly, fecal samples were manually collected from each animal’s anus and dispensed in 2-mL sterile tubes. The fecal samples were immediately immersed in liquid nitrogen and stored at −80 °C until DNA extraction. Sow estrus behaviors had been recorded since the weaning day, as described in our previous study [14]. Those sows for which estrus behaviors and symptoms had not been observed for more than 14 d since the weaning day were defined as non-return sows. All experimental sows were divided into two groups according to the interval from weaning to estrus return: the normal return group (167 sows in the discovery cohort and 18 sows in the validation cohort), in which the sows returned to estrus within 7 d after weaning, and the non-return group (40 sows from the discovery cohort and 11 sows from the validation cohort), in which the sows failed to return to estrus within 14 d after weaning.

### Microbial DNA extraction

Microbial DNA from fecal samples was extracted using an E.Z.N.A.® soil DNA kit (M9636-02, Omega Bio-Tek, Norcross, GA, USA) according to the manufacturer’s manuals. Microbial DNA quality was checked using 1.0% agarose gel electrophoresis (JY600C, JUNYI, Beijing, China), and the concentration and purity of extracted DNA were determined with a NanoDrop® ND-2000 spectrophotometer (ND2000, Thermo Scientific Inc., Waltham, MA, USA). All DNA samples were stored at −80 °C until use.

### 16S rRNA gene sequencing and bioinformatic analysis

PCR amplification was performed for the V3-V4 hypervariable region of the 16S rRNA gene using the primers 338F (5'-ACTCCTACGGGAGGCAGCAG-3') and 806R (5'-GGACTACHVGGGTWTCTAAT-3') by an ABI GeneAmp® 9700 PCR thermocycler (ABI-97, ABI, Los Angeles, CA, USA) for 207 fecal microbiota DNA samples. The conditions for the PCR amplification were described in our previous study [14]. The purified PCR products were used to construct libraries and were sequenced on an Illumina MiSeq PE300 platform with a paired-end strategy (SY-410-1003, Illumina, San Diego, CA, USA) according to standard protocols by Majorbio Bio-Pharm Technology Co. Ltd. (Shanghai, China). After removing the barcodes, all sequence reads of tested samples were processed using the QIIME2 Core 2021.11 pipeline [17] with the following parameters: the demultiplexed paired-reads were merged using the q2-vsearch plugin (via vsearch join-pairs), and raw reads were filtered based on the quality scores with the q2-quality-filter plugin using default parameters. The high-quality reads were denoised using the Deblur algorithm [18] with recommended parameters except –p-trim-length 390. Finally, a feature table of amplicon sequence variants (ASVs) was obtained for downstream analyses. ASVs identified as the sequences of mitochondrial genes were filtered. An additional step was employed to remove the samples with a total number of less than 4,000 reads. The sequencing depth of fecal samples was rarefied to 10,879 tags to avoid the effect of uneven sequencing depth on α- and β-diversity of the microbial composition. Taxonomic assignment of ASVs was performed based on the Silva database using the q2-feature-classifier plugin. The pre-trained Naive Bayes taxonomy classifier for the V3-V4 region (silva-138-99-seqs-338-806-classifier.qza) was used in the classification.

### Shotgun metagenomic sequencing and bioinformatic analysis

A total of 85 fecal DNA samples from the discovery cohort, including 45 samples from normal return sows and 40 samples from non-return sows, and 29 fecal DNA samples from the validation cohort, including 18 samples from normal return sows and 11 samples from non-return sows, were used for shotgun metagenomic sequencing analysis. These samples covered all non-return sows that we could obtain and a similar number of normal return sows that were selected randomly from both cohorts. DNA samples were fragmented to an average size of about 400 bp using Covaris M220 (Gene Company Limited, Hong Kong, China). Paired-end libraries were constructed using NEXTFLEX Rapid DNA-Seq (Bioo Scientific, Austin, TX, USA). Adapters containing the full complement of sequencing primer hybridization sites were ligated to the blunt ends of fragments. Paired-end sequencing was performed on an Illumina NovaSeq 6000 platform (Illumina, San Diego, CA, USA) at Majorbio Bio-Pharm Technology Co., Ltd. (Shanghai, China) using NovaSeq Reagent Kits according to the manufacturer’s protocol. Adapters, low-quality reads, and host genomic sequences were filtered from the raw reads before assembly using the software fastp (v0.20.0, –cut_by_quality3 -W 4 -M 20 -n 5 -c -l 50 -w 3) [19] and bwa-mem2 (v2.2.1, -t 16 -T 30) [20]. The clean reads were used to conduct single-sample assembly using MEGAHIT (v1.1.3, –min-count 2 –k-min 27 –k-max 127 –k-step 20 –min-contig-len 1000) [21]. The gene prediction of assembled contigs was performed using Prodigal (v2.6.3) [22]. Complete genes (containing both a start and stop codon) were retained and used to construct a non-redundant gene catalog containing 3,990,509 genes at the 90% identity level by CD-HIT (v4.7, -c 0.9 -s 0.8) [23]. The protein sequences of non-redundant genes were then aligned to the Uniprot TrEMBL database by DIAMOND (v2.0.8) [24] with e-values ≤ 1e−5. Taxonomic classification of predicted genes was performed using the BASTA (v1.3.2.3, -l 25 -i 80 -e 0.00001 -p 60) software based on the Lowest Common Ancestor algorithm [25]. All predicted genes were functionally annotated using the KEGG (Kyoto Encyclopedia of Genes and Genomes) and dbCAN databases (HMMdb V9) with the DIAMOND (v2.0.8) and HMMER programs (v3.1b2), respectively. Meanwhile, KOBAS (v3.0) [26] was used to retrieve KO annotation and identify the pathways with high frequency as well as statistically different enrichments from the annotation results. The abundance of predicted genes was estimated using featureCounts (v1.6.2, -p) [27]. The abundances were normalized to fragments per kilobase of gene sequence per million reads mapped (FPKM) [28]. The abundances of microbial taxa and KEGG pathways were quantified by summing the abundances of genes belonging to each category [29].

### Construction of co-abundance groups (CAGs) of bacterial species with metagenomic sequencing data

The correlations among gut bacterial species obtained from shotgun metagenomic sequencing data were assessed in both discovery and validation cohorts based on the sparse correlations for compositional data (SparCC) algorithm [30] implemented in FastSpar (v1.0.0) [31]. A total of 1,000 bootstrap samples were generated with the command fastspar_bootstrap, and the correlation matrices of the resampled data matrices were calculated with 50 iterations. From 1,000 bootstrap correlations, *P*-values were then calculated using 1,000 permutations with the command fastspar_pvalues. The correlation values were converted to a correlation distance (1 – the correlation value), and the species were clustered using the Ward clustering algorithm via the WGCNA package [32] in R program. Similar clusters were subsequently merged if the correlation coefficient between the CAG’s eigenvectors exceeded 0.8. The CAG network was visualized via Cytoscape (v3.7.1).

### Widely targeted metabolome and lipidomics measurements of feces samples

The fecal samples were thawed on ice. For widely targeted metabolome measurement, 50 mg (± 1 mg) was taken from each fecal sample and homogenized with 500 μL of ice-cold methanol/water (70%, v/v) with an internal standard. The homogenate was vortexed for 3 min, sonicated for 10 min in an ice water bath, and then further vortexed for 1 min. The mixtures were centrifuged at 12,000 r/min at 4 °C for 10 min. Subsequently, 250 μL of the supernatant was pipetted out and centrifuged again at 12,000 r/min for 5 min at 4 °C before on-board analysis. The sample extracts were analyzed using an LC–ESI–MS/MS system (UPLC: LC-30A, Shimadzu, Kyoto, Japan; ESI–MS/MS system: QTRAP 6500+ , SCIEX, Foster City, CA, USA). For each sample, 2 μL of extracted supernatant was injected into a Waters Acquity UPLC HSS T3 C18 column (1.8 μm × 2.1 mm × 100 mm, 186003539, Waters, Taunton, MA, USA) at 40 °C. The flow rate was set at 0.4 mL/min. Water containing 0.1% formic acid (A) and acetonitrile containing 0.1% formic acid (B) were used as the mobile phase. Linear ion trap (LIT) and triple quadrupole (QQQ) scans were acquired on a triple quadrupole-linear ion trap mass spectrometer (TripleTOF 6600+ , SCIEX, Foster City, CA, USA) equipped with an ESI Turbo Ion-Spray interface, operating in positive and negative ion mode, and controlled by the Analyst software (v1.6.3, SCIEX). The ESI source temperature and ion spray voltage (IS) were set at 500 °C and 5,500 V for positive ion mode, and 500 °C and −4,500 V for negative ion mode. Meanwhile, the ion source gas 1 (GS1), gas 2 (GS2), and curtain gas (CUR) were set at 50, 50, and 25 psi, respectively. Instrument tuning and mass calibration were performed with 10 and 100 μmol/L polypropylene glycol solutions in QQQ and LIT modes, respectively.

For fecal lipidomics measurements, 20 mg of feces was taken from each sample and homogenized with 1 mL of methyl-tert-butyl ether (MTBE)/methanol (3:1, v/v) with the internal standard mixture and a steel ball. After removing the steel ball, the mixture was vortexed for 15 min. Subsequently, 200 μL of water was added to the mixture and vortexed for 1 min. The mixture was centrifuged at 12,000 r/min at 4 °C for 10 min. A total of 300 μL of the supernatant was pipetted from each sample and freeze-dried. The powder was then dissolved in 200 μL of mobile phase B. The lipidomics extracts were analyzed using an LC–ESI–MS/MS system (UPLC: SCIEX ExionLC AD system; MS: SCIEX QTRAP 6500+ system). A total of 2 μL of lipidomics extracts for each sample was injected into a Thermo Accucore™ C30 column (2.6 μm × 2.1 mm × 100 mm) (27826–102130, Thermo Scientific, Waltham, MA, USA) at 45 °C. The flow rate was set at 0.35 mL/min. Acetonitrile/water (60/40, v/v) containing 0.1% formic acid (A) and acetonitrile/isopropanol (10/90, v/v) containing 0.1% formic acid (B) were used as the mobile phase. The ion source GS1, GS2, and CUR were set at 45, 55, and 35 psi, respectively. The parameters of ESI source temperature, IS, instrument tuning, and mass calibration were the same as those for the metabolome measurements.

The mass spectrum data were acquired by Analyst software (v1.6.3, SCIEX). The repeatability of the extraction and detection of metabolites were determined by total ion current and multi-peak diagram in the multiple reaction monitoring (MRM) mode. The qualitative analysis of secondary general data and the identification of metabolites were carried out according to the retention time (RT) and mass-to-charge ratio (*m/z*) based on both an in-house MWDB database (Metware Biotechnology Co., Ltd., Wuhan, China) and several publicly available databases, including massbank (http://www.massbank.jp/), Metlin [33], HMDB [34], and knapsack (http://kanaya.naist.jp/knapsack/). Quality control (QC) samples were prepared by mixing 10 μL of each sample. One QC sample was injected per 10 samples following the running order. Metabolite quantification was accomplished using MRM of triple quadrupole mass spectrometry. The chromatographic peaks of the mass spectrum files were integrated and calibrated in the QTRAP® system. The peak area of each chromatographic peak represented the relative concentration of the corresponding metabolite. We calculated the coefficient of variation (CV) values of the metabolites in the QC samples and removed those metabolites whose CV values were greater than 0.5.

### Statistical analysis

#### Comparisons of the α- and β-diversity of the gut microbiome between normal return and non-return sows

Using the 16S rRNA gene sequencing data, the α-diversity indices of observed species, Chao1, ACE, Shannon, Simpson, and Evenness were calculated and visualized with the MicrobiotaProcess R package (v1.7.8). The α-diversity of the gut microbial composition between normal return and non-return sows was compared by a Wilcoxon rank-sum test. The β-diversity based on the Bray–Curtis distance was analyzed using the q2-diversity plugin in QIIME2. A principal coordinate analysis (PCoA) was performed based on the microbial dissimilarity between the two groups and visualized using R software to assess the differences in the microbiota composition between normal return and non-return sows. 

For the metagenomic sequencing data, the α-diversity (observed species, Chao, ACE, and Shannon indices) and the β-diversity based on the Bray–Curtis distance were estimated via the Vegan package for R software (v4.1.2). A Wilcoxon rank-sum test was used to compare the α- and β-diversity of the gut microbiome between normal return and non-return sows. The results were visualized using the ggplot2 package for R.

#### Identification of microbial taxa, CAGs, and functional capacities showing different abundances between normal return and non-return sows

The mp_diff_analysis() function of the MicrobiotaProcess R package (v1.7.8) was used to identify the ASVs and taxa whose abundances varied significantly between normal return and non-return sows at the significance thresholds of false discovery rate (FDR) < 0.05 and linear discriminant analysis (LDA) score > 3.0 using the 16S rRNA gene sequencing data. Differential CAGs between normal and non-return sows were identified by a Wilcoxon rank-sum test at the significance threshold of *P* < 0.05. The linear discriminate analysis effect size (LEfSe) algorithm [35] was used to identify microbial taxa showing significant differences in the abundances between normal return and non-return sows at the significance threshold of *P* < 0.05 and LDA score > 2 using metagenomic sequencing data. The relative abundance of *P. copri* in each tested sample was defined as the number of reads aligned to the *P. copri* genome normalized by the total number of reads in that sample and the genome size. The Wilcoxon rank-sum test was also performed to discriminate the functional capacities of the gut microbiome having significantly different abundances between two sow groups at the threshold of FDR < 0.05.

#### Data analysis of widely targeted metabolome and lipidomics

Orthogonal partial least squares discriminant analysis (OPLS-DA) was performed to evaluate the differences of widely targeted metabolome profiles between normal return and non-return sows using the online MetaboAnalyst (v5.0) [36] with default parameters. The differential metabolites, lipid molecules, and metabolite modules were first identified by univariate analysis with Student's* t*-test at the significance threshold of FDR < 0.05. Then, the differential metabolites and lipid molecules with FDR < 0.05 and the absolute value of log_2_ (fold change) > 1 were visualized by a volcano plot. Differential candidates of metabolites and lipid molecules with FDR < 0.05 and variable importance in projection (VIP) > 1 from the OPLS-DA model were clustered into co-abundance modules using the WGCNA R package. Signed and weighted co-abundance correlation networks of metabolites were constructed after log_2_(*x* + 1) transformation of the concentrations of metabolites across 85 experimental sows. According to the scale-free topological criterion (*R*^2^ = 0.9), we chose the soft threshold of β = 16 for the correlations of widely targeted metabolites and β = 28 for lipid molecules. Metabolite clusters were identified with the dynamic hybrid tree-cutting algorithm at the threshold for deepSplit of 4 and minimum cluster sizes of 5 and 3 for widely targeted metabolites and lipid molecules, respectively. The metabolites that did not fit the clustering criteria were recruited in a group named ‘remaining’. The eigenvector of each module was selected as the representative value of its profile. The modules with similarity of eigengenes > 0.8 were merged. The clusters of widely targeted metabolites and lipid molecules were labeled M01–M35 and L01, L03–L22, respectively.

#### Correlation analysis between the gut microbiome and fecal metabolome

To assess the relationships between the changes in gut microbiota and the shifts in fecal metabolites, Spearman correlation analysis was performed between differential gut microbial taxa and fecal metabolites, between differential CAGs and metabolite modules, and between differential gut microbial taxa and hormones and hormone-related compounds. We computed *P*-values and applied the Benjamini–Hochberg method to control for the FDR. The correlations with FDR < 0.05 were visualized with the ggplot2 and ComplexHeatmap packages for R software (v4.1.2).

#### Random Forest model for identifying the biomarkers that could be used to classify return and non-return sows

To distinguish return and non-return sows, a random forest classification model was constructed based on fecal CAGs and metabolite modules using k-fold cross-validation, where k was the number of samples. The optimal combination was chosen at the lowest cross-validation error. The efficiency of classification was assessed using receiver operating characteristic (ROC) curves, and the area under the ROC curve (AUC) was measured using the pROC package for R software (v4.1.2).

## Results

### Gut microbial composition of sows after weaning and identification of the gut microbial taxa associated with estrus return of post-weaning sows

A 16S rRNA gene sequencing analysis was performed for a total of 207 fecal samples to provide a comprehensive understanding of the composition of the gut microbiota in sows after weaning. On average, 16,027 clean tags were obtained for each sample, and these tags were clustered into 4,880 ASVs using a deblur denoising pipeline. Rarefaction curves based on the α-diversity indices indicated that the number of sequence reads for each fecal sample was sufficient for further analysis (Fig. S2A). After the taxonomic annotation of ASVs, we obtained a profile of the taxonomic composition of the gut microbiota. The gut microbial community was dominated by Firmicutes (77.51%), Bacteroidota (13.2%), Spirochaetota (5.85%), and Proteobacteria (2.15%). At the genus level, *Clostridium_sensu_stricto_1* (15.07%), *Terrisporobacter (*11.09%), *Treponema* (5.77%), *Turicibacter* (5.42%), and *Lactobacillus* (5.26%) were ranked in the top 5 in relative abundances (Fig. S2B). We evaluated the effect of sow parities on the gut microbial composition because experimental sows were at different parities. The results showed that the parity only accounted for 0.17% of the variation of the gut microbial composition, suggesting no significant effect (Fig. S3).

All 207 experimental sows were divided into a normal return group (167 fecal samples) and a non-return group (40 samples) according to the interval from weaning to estrus return (Methods). Although there was no significant difference in the α-diversity of gut microbiota between the two sow groups, the richness of observed taxa, Chao1, ACE, and Shannon indices in non-return sows were higher than those in normal return sows (Fig. S4A). Meanwhile, significant shifts in gut microbiota composition between normal return and non-return sows were observed from the PCoA based on the Bray–Curtis distance (Fig. S4B and C).

We further identified bacterial taxa having significantly differential abundances between the two groups. At the genus level, genera from *Muribaculaceae*, *Lachnospiraceae_XPB1014_group*, and *Prevotella* were significantly enriched in normal return sows, while 4 genera had significantly higher abundances in non-return sows, including *Streptococcus*, *Bacteroides, Family_XIII_AD3011_group* (belonging to Anaerovoracaceae) and *Bifidobacterium* (Fig. S4E, Table S2). At the ASV level, 15 ASVs showed significantly differential abundances between the two sow groups. ASV0014:*s_Lactobacillus_johnsonii*, ASV0302:*g_Lachnospiraceae_XPB1014*, ASV0714:*g_Muribaculaceae*, and ASV0242:*g_Muribaculaceae* were significantly enriched in normal sows, whereas the abundances of 11 ASVs were significantly higher in non-return sows, including ASV0109:*g_Streptococcus*, ASV0048:*g_Clostridium_sensu_stricto_1*, ASV0088:*s_Clostridium_septicum*, ASV0059:*g_Family_XIII_AD3011_group*, ASV0201:*g_Clostridia_UCG014*, ASV0310:*s_Bacteroides_fragilis*, and ASV0384:*s_Bifidobacterium_pseudolongum* (Fig. S4D, Table S2).

**Fig. 1:**
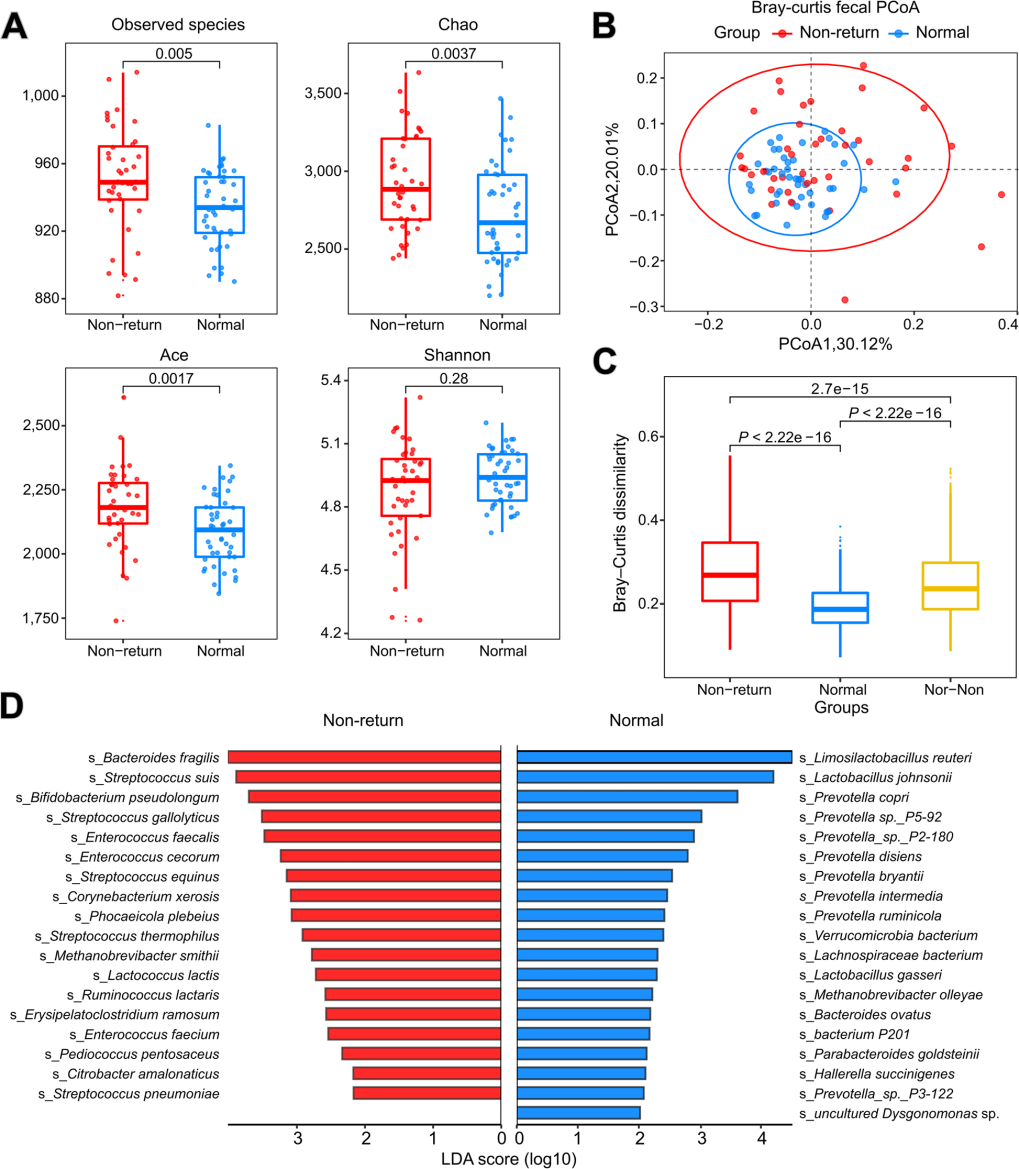
Comparison of the diversity of gut microbiota and identification of gut bacterial species showing differential abundances between normal and non-return sows. **A** Comparison of the α-diversity of gut microbiota. **B** Comparison of the gut microbial composition between normal return and non-return sows by principal coordinate analysis (PCoA) based on the Bray–Curtis distance. **C** Boxplot of the Bray–Curtis dissimilarity of gut microbiota between subjects within and between each group. Comparison was performed by Wilcoxon rank-sum test at the significance level of *P* < 0.05. **D** Differential bacterial species between normal return and non-return sows with metagenomic sequencing data. The significance threshold was set at LDA score > 2 and *P* < 0.05

Shotgun metagenomic sequencing was performed for 85 fecal samples, with 45 samples from normal return sows and 40 samples from non-return sows (Table S3**)**. There was no significant difference in gene richness between the two sow groups (Fig. S5A). The α-diversity indices including observed species, Chao, and ACE in normal return sows were significantly lower than those in non-return sows, although the difference in the Shannon index did not achieve a significant level (Fig. 1A). The β-diversity of gut microbial composition was also significantly different between normal return and non-return sows (Fig. 1B and C).

*Streptococcus*, *Bacteroides*, *Enterococcus*, and *Bifidobacterium* were more abundant in non-return sows, whereas *Limosilactobacillus*, *Prevotella*, and *Treponema* had higher abundances in normal return sows (Fig. S5C). The LEfSe analysis further identified 37 bacterial species showing distinct abundances between the two sow groups. Among these species, 19 species were significantly enriched in normal sows, most of which belonged to *Lactobacillus* and *Prevotella*. The relative abundances of 18 species were significantly higher in non-return sows, including *B. fragilis*, *B. pseudolongum*, and several species belonging to *Streptococcus* and *Enterococcus* (Fig. 1D, Table S4). This result agreed with the findings based on the 16S rRNA gene sequencing data, suggesting that these species should be treated as potential microbial biomarkers to distinguish normal return and non-return sows.

To further validate the association analysis results, we performed a multivariate logistic regression to examine the relationship between gut microbial species and estrus return in experimental sows by adopting the models with and without adjusting for the effect of parity. Notably, the estrus return-associated bacterial species were mostly consistent with those identified in the LEfSe analysis. Furthermore, the parities showed no effect on the identification of estrus return-associated bacterial species (Table S5).

### Co-abundance group analysis identified the hub bacterial species associated with estrus return of post-weaning sows

**Fig. 2:**
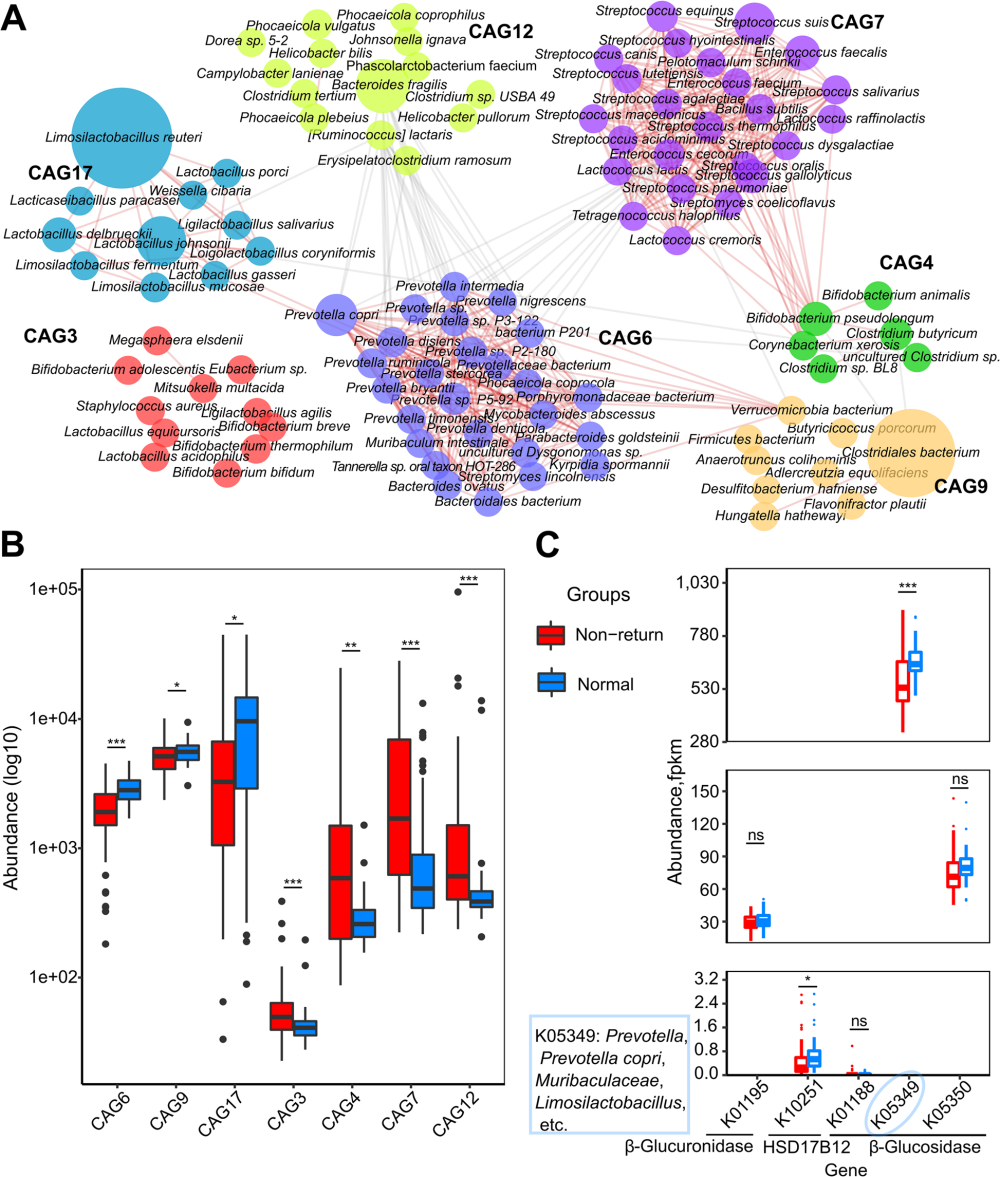
Co-abundance groups (CAGs) of bacterial species showing different abundances between normal and non-return sows and comparing the abundances of bacterial genes participating in the deconjugation of estrogen. **A** The network diagram of 7 CAGs showing different enrichments between normal return and non-return sows. The size of the nodes represents the abundance of bacterial species. The thickness of the connections between nodes indicates the weight of the correlation coefficient between species. Lines were drawn only when its correlation coefficient was greater than 0.5 and *P* < 0.05. The red lines represent a positive correlation and the gray lines represent a negative correlation. The colors of the nodes represent different CAGs. **B** Boxplots show the differential abundances of seven CAGs between two sow groups. Wilcoxon rank-sum test was performed for comparison analysis. ^*^P < 0.05, ^**^P < 0.01, ^***^P < 0.001. **C** The comparisons of the abundances of β-glucuronidase, β-glucosidase, and hydroxysteroid dehydrogenases that participate in the deconjugation of estrogen. Wilcoxon rank-sum test was used for the comparison, ns, FDR > 0.05, ^*^FDR < 0.05, ^***^FDR < 0.001. *Muribaculaceae*, *Prevotella*, *Prevotella copri*, and *Limosilactobacillus* carried the K05349 gene that was annotated to β-glucosidase in their genomes

A co-abundance network was constructed based on the SparCC algorithm with 540 bacterial species obtained from metagenomic sequencing analysis. A total of 25 CAGs were obtained and compared between the two sow groups (Table S6). Seven CAGs showed significantly different enrichment between the groups (*P* < 0.05) (Fig. 2B, Table S8). Of these, CAG6, CAG17, and CAG9 were enriched in normal sows. Notably, CAG6 comprised 12 species from *Prevotella*, including *P. copri* and *P. bryantii*. The species from *Limosilactobacillus* and *Lactobacillus* were included in CAG17. CAG9 was largely composed of *Clostridiales bacterium* and *Verrucomicrobia bacterium*. Combining their abundances in the gut microbiome and the number of connections in the networks, *L. reuteri*, *P. copri*, and *Verrucomicrobia bacterium* were the hub species enriched in normal sows. Conversely, the abundances of CAG7, CAG12, CAG4, and CAG3 were significantly increased in non-return sows. The species included in each of these CAGs are shown in Fig. 2A. *B. fragilis*, *S. suis*, and *B. pseudolongum* were the hub species enriched in the gut of non-return sows. Positive correlations were identified among CAG6, CAG17, and CAG9 as well as between CAG7 and CAG4. These results suggested that the CAGs may affect the estrus return of sows after weaning by synergistic regulation. Notably, CAG6 was negatively correlated with CAG12, CAG4, and CAG7 (Fig. 2A), suggesting competition between normal return-associated bacteria and non-return-associated bacteria in the gut microecosystem.

### Validating the estrus return-associated bacterial species in an independent validation sow cohort

Fecal samples of 18 normal return and 11 non-return sows from an independent validation cohort were subjected to shotgun metagenomic sequencing to further confirm the differential bacterial taxa between normal return and non-return sows (Table S3). The comparison results for the α- and β-diversity between the two sow groups are shown in Fig. S6A–C. At the species level, a total of 27 bacterial species showed different abundances between normal and non-return sows. Of these, 11 species were significantly enriched in normal sows, most of which belonged to *Lactobacillus*. The relative abundances of 16 species including *E. siraeum*, *B. pseudolongum*, and several species belonging to *Streptococcus* were significantly higher in non-return sows (Fig. S6D). We note that among these differentially abundant species, *L. johnsonii*, *L. gasseri*, and *Verrucomicrobia bacterium* that were enriched in normal sows, and the species *B. pseudolongum*, *S. suis*, *R. lactaris*, and *S. equinus* enriched in non-return sows were repeated between the discovery and validation cohorts. At the CAG level, 7 out of 32 CAGs derived from 495 species in the validation cohort showed significantly different abundances between normal-return and non-return sows (Fig. S7B and Table S8). Of these, CAG3 was largely composed of *Bifidobacterium* spp. including *B. pseudolongum* and *B. bifidum* were significantly enriched in non-return sows. In contrast, CAG17 composed of *Prevotella* spp*.* and *Muribaculaceae bacterium*, and CAG23 including the species from *Limosilactobacillus* and *Lactobacillus* were significantly enriched in normal return sows (Fig. S7A and Table S7). These findings were also consistent with those obtained in the discovery cohort.

### Significant changes in functional capacities of the gut microbiome between normal return and non-return sows

The gut microbial genes were aligned to the KEGG database to explore differential KEGG pathways between normal return and non-return sows. A total of 1,823 differential KEGG orthology genes (KO genes) were identified between the two sow groups in the discovery cohort (FDR < 0.05), and these differential KO genes were involved in 233 functional pathways (Table S9). Of these, 34 pathways were significantly enriched in non-return sows, including ABC transporter, pyrimidine metabolism, purine metabolism, and steroid degradation. while 199 KEGG pathways exhibited significantly higher abundance in normal sows, including energy metabolism, lipid metabolism (e.g., steroid hormone biosynthesis and glycerophospholipid metabolism), carbohydrate metabolism (TCA cycle and glycolysis/gluconeogenesis), glycan biosynthesis and metabolism (lipopolysaccharide biosynthesis), endocrine system (estrogen signaling pathway, GnRH signaling pathway, and ovarian steroidogenesis), and digestive system (vitamin digestion and absorption) (Table S9). Significant changes in potential functional capacities of the gut microbiome between normal return and non-return sows were confirmed in the validation sow cohort. A total of 33 KEGG pathways showed significantly higher abundance in normal return sows. Twenty-three out of these 33 differential KEGG pathways were also identified in the discovery cohort, including steroid hormone biosynthesis, glycerophospholipid metabolism, glycan biosynthesis and metabolism (lipopolysaccharide biosynthesis), glycolysis/gluconeogenesis, and the estrogen signaling pathway (Table S10). However, several immune and disease-related KEGG pathways were enriched in non-return sows. Notably, microbial genes having higher abundances in normal sows were enriched in the pathways of steroid hormone biosynthesis, estrogen signaling pathway, GnRH signaling pathway, and ovarian steroidogenesis, further implying that gut microbiota may play an important role in the process of estrus return in post-weaning sows.

We compared the abundances of β-glucuronidase, β-glucosidase, and HSD in more detail, all of which participate in the deconjugation and reuptake of estrogens, between the two sow groups. In normal return sows of the discovery cohort, β-glucosidase (K05349) and HSD17B12 (K10251) had significantly higher abundances in the gut microbiome (FDR = 4.37e–04 and 0.03), and the abundance of β-glucuronidase was also higher, although it did not achieve a significant level (FDR = 0.24) (Fig. 2C). Specifically, some taxa significantly enriched in normal sows, such as *Muribaculaceae*, *Prevotella* (especially *P. copri*) and *Limosilactobacillus*, carried β-glucosidase in their genomes (Fig. 2C and S5D). Moreover, significantly positive correlations were observed in relative abundance between these gut microbes and β-glucosidase (K05349), except for *Limosilactobacillus* (Fig. S5E). *P. copri* was the hub species in CAG6 that was enriched in normal sows (Fig. 2A). We isolated and cultured *P. copri *in vitro from pig fecal samples in our previous study [37]. Here, the β-glucosidase (K05349) was indeed found in the genome of the *P. copri* isolate. The *P*. *copri* isolate showed significantly higher abundance in normal sows with metagenomic sequencing data (Fig. S5B). Importantly, a significant positive correlation was observed between the abundances of the *P. copri* isolate and β-glucosidase (*R*^2^ = 0.60, *P* = 2.28e–11). Consistently, the gut microbiome of normal return sows in the validation cohort also had higher abundances of β-glucuronidase, β-glucosidase, and hydroxysteroid dehydrogenases compared to the non-return sows (Fig. S7D), although the differences did not achieve a significant level due to the smaller sample size. These findings suggested that the alterations in the gut microbiome may cause changes in estrogen levels in the host. This could be one of the reasons leading to estrous cycle disorders in sows.

### Alterations in fecal metabolome profiles between normal return and non-return sows

**Fig. 3:**
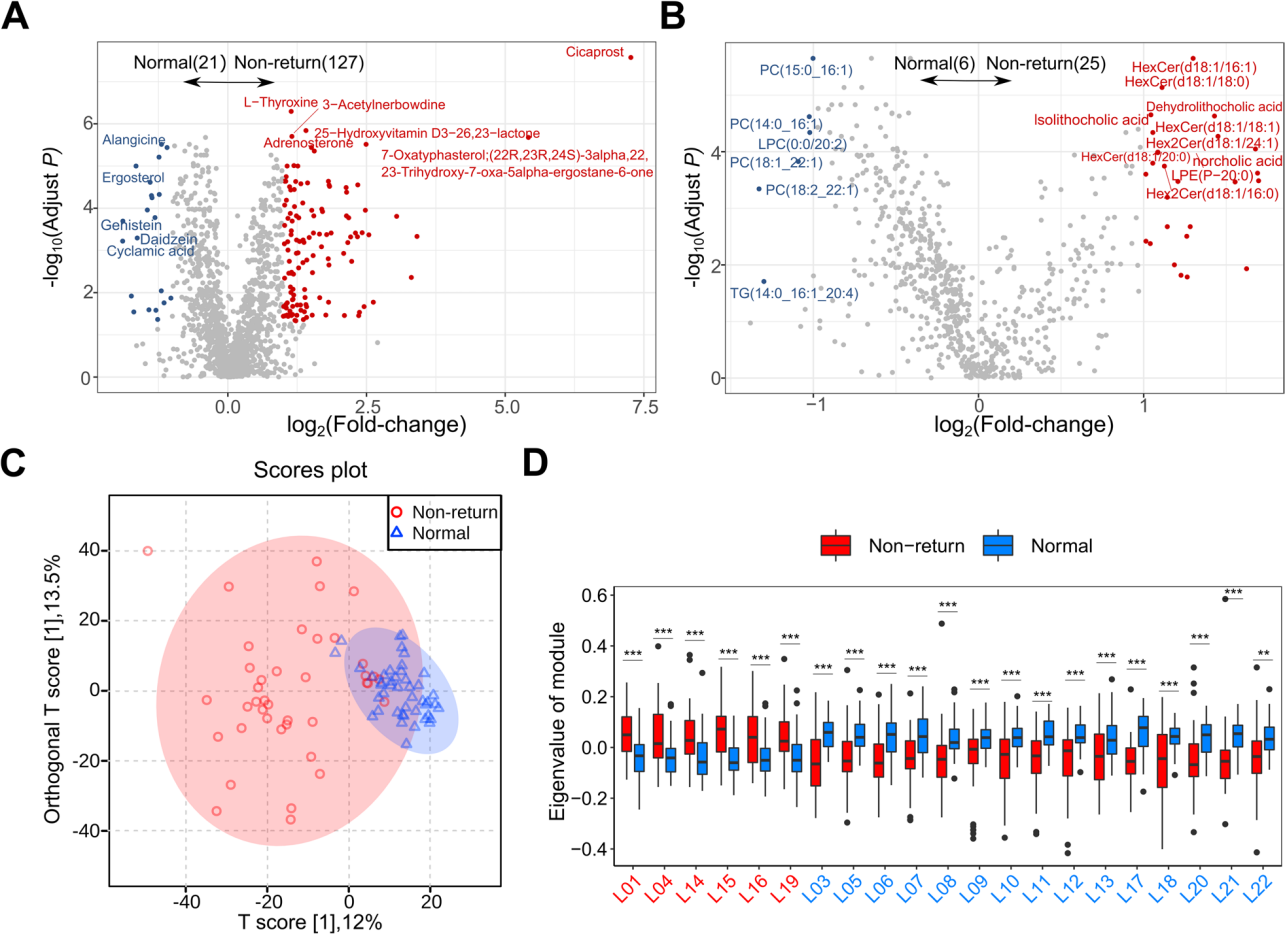
Alterations in fecal metabolome profiles between normal return and non-return sows. Volcano plots identifying the differential fecal metabolites by widely targeted metabolome (**A**) and lipidome (**B**), only the metabolites with FDR less than 0.05 and the absolute value of log_2_ (fold change) greater than 1 are colored, red represents the metabolites enriched in non-return sows and blue represents those metabolites enriched in normal sows. **C** OPLS-DA diagram of fecal metabolome profile shows that metabolite profiles in feces samples of normal sows were clearly separated from that of non-return sows. Boxplots show differential metabolite modules between normal return and non-return sows. **D** Student's* t*-test identified a total of 21 differential lipid molecule modules. ^*^FDR < 0.05, ^**^FDR < 0.01, and ^***^FDR < 0.001

Widely targeted metabolome and lipidome measurements were performed on 85 fecal samples as described above to compare the metabolite profiles between normal return and non-return sows in the discovery cohort. A total of 1,863 metabolites and 718 lipid molecules were obtained and employed for further analysis. We observed that the metabolite profiles in normal return sows were clearly separated from those of non-return sows by OPLS-DA (Fig. 3C). Compared with normal return sows, non-return sows displayed increased concentrations of 127 metabolites and decreased concentrations of 21 metabolites in the widely targeted metabolome data (Fig. 3A, Table S11). Daidzein, genistein, ergosterol, and 3-*O*-*p*-coumaroylquinic acid had significantly higher concentrations in normal sows. However, cicaprost, bolasterone, 5-pregnen-3β-ol-20-one, allopregnan-20alpha-ol-3-one, adrenosterone, and phosphate sugars including D-fructose-6-phosphate-disodium salt and *N*-acetylglucosamine-1-phosphate showed significantly higher concentrations in non-return sows. Meanwhile, a total of 31 differentially abundant lipid molecules were identified between two sow groups, including 25 lipid molecules enriched in non-return sows and 6 lipid metabolites having higher concentrations in normal sows (Fig. 3B and Table S11).

**Fig. 4:**
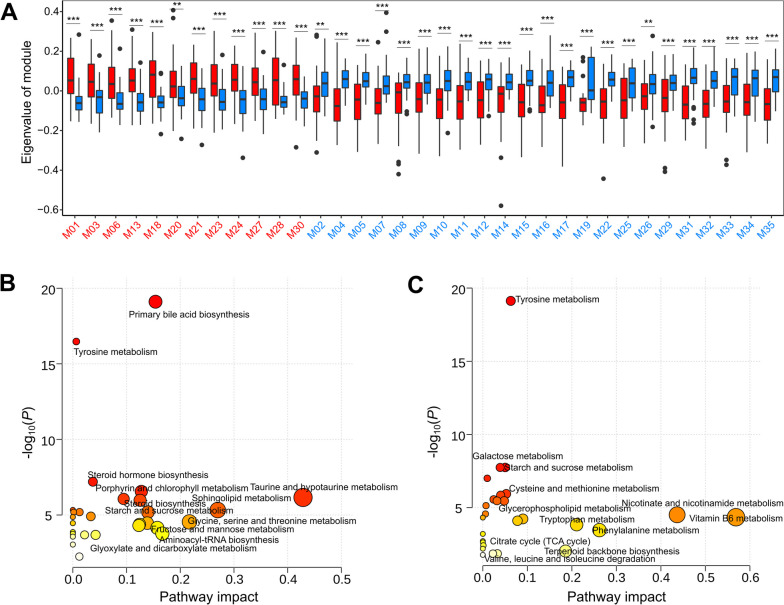
Alterations in fecal metabolome profiles between normal return and non-return sows. **A** 35 differential metabolite modules identified by Student's *t*-test. ^*^FDR < 0.05, ^**^FDR < 0.01, ^***^FDR < 0.001. The *y*-axis indicates the eigenvector of each module as the representative value of its metabolite profile. **B** and **C** The KEGG pathways enriched by differential fecal metabolite features (including both lipid molecules and metabolites) in non-return (**B**) and normal return sows (**C**). The size and color of the dots indicate the value of the KEGG pathway impact and the enrichment significance of the pathways, respectively

Considering the complex relationships among fecal metabolites, and to further explore the biological function patterns of different metabolites, all differential compounds, including 630 metabolites from the widely targeted metabolome data and 274 lipid molecules from lipidome data detected by OPLS-DA models (VIP > 1, FDR < 0.05), were clustered into 35 metabolite modules and 21 lipid molecule modules (Table S12). Among the 21 lipid modules, 6 modules containing 68 lipid molecules were enriched in non-return sows, while 15 modules containing 163 lipid molecules were enriched in normal sows (Fig. 3D, Table S12 and S13). Among the 35 metabolite modules, 12 modules including 296 metabolites were enriched in non-return sows, while 23 modules containing 281 metabolites showed enrichment in normal sows (Fig. 4A, Table S12 and S13). We then performed a KEGG enrichment analysis for all differential metabolites. The metabolites having higher concentrations in non-return sows were enriched in primary bile acid biosynthesis, tyrosine metabolism, steroid hormone biosynthesis, taurine and hypotaurine metabolism, and steroid biosynthesis (Fig. 4B). However, the metabolites having higher concentrations in normal return sows showed enrichment in the pathways of tyrosine metabolism, galactose metabolism, butyrate metabolism, nicotinic acid and nicotinamide metabolism, vitamin B_6_ metabolism, steroid hormone biosynthesis, and tryptophan metabolism (Fig. 4C). Notably, when we focused on the metabolites enriched in the pathways involved in hormones and hormone-related compounds, androgens, progestogens, and related compounds including testosterone, oxandrolone, adrenosterone, bolasterone, testosterone cypionate, allopregnan-20alpha-ol-3-one, and 5-pregnen-3β-ol-20-one were enriched in non-return sows. However, estrogen-related compounds such as estradiol-17 phenylpropionate, estrone 3-sulfate, phytoestrogens (including genistein and daidzein), and solasodine, which has an anti-androgenic effect, were significantly enriched in normal return sows. These results indicated that the level of hormones and metabolites were significantly different between the two sow groups, and thus the changes in hormones may influence estrus return of post-weaning sows.

### Shifts in fecal metabolites were associated with the changes in the gut microbiome

**Fig. 5:**
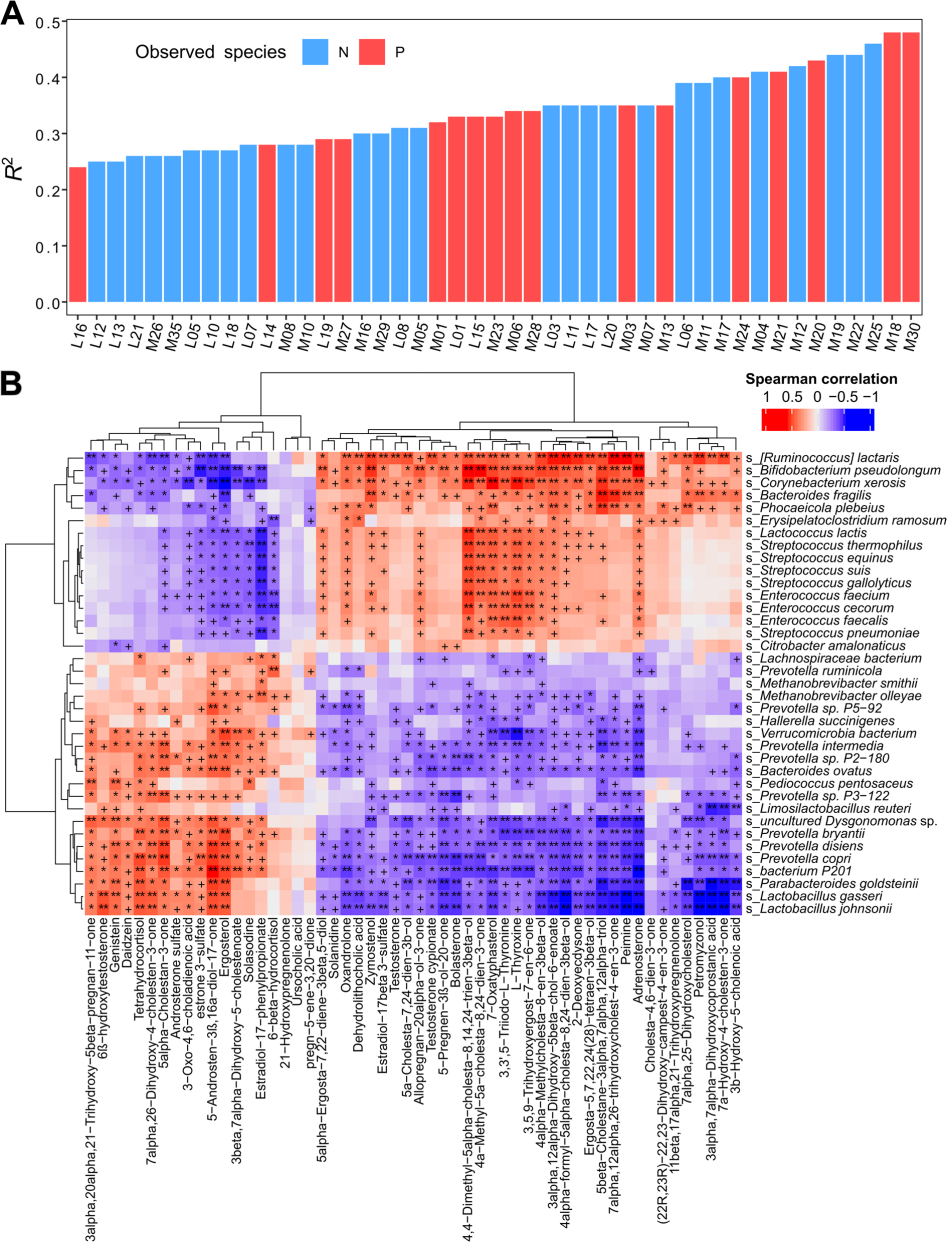
The interrelationship between gut microbiota and fecal metabolite profile by integrating fecal metagenomic and metabolome data. **A** The association between the α-diversity index of observed species and metabolite modules. Red represents a positive correlation and blue represents a negative correlation. Only the correlations with FDR < 0.05 were displayed. **B** The heatmap depicts the relationships between differential bacterial species, and fecal hormones and hormone related compounds. Spearman correlation coefficients were used. Red represents positive correlations and blue represents negative correlations. The stars in the grids represent the significance threshold: ^+^FDR < 0.05, ^*^FDR < 0.01, ^**^FDR < 0.001

We evaluated the associations between the changes in the gut microbiome and the shifts in fecal metabolites to highlight the possible mechanism of gut microbiome influencing sow estrus return. At the significance threshold of FDR < 0.05, the α-diversity (observed species) of the gut microbiota was significantly associated with 45 differential metabolite modules, with 17 metabolite modules and 28 lipid molecule modules. Specifically, M30 and M18 modules that contained hormones and hormone-related compounds showed the strongest positive correlations, indicating a significant relationship between the composition of the gut microbiome and hormones and hormone-related compounds (Fig. 5A). Subsequently, significant relationships were discovered between 7 differential CAGs and 56 differential metabolite modules (21 lipid molecule modules and 35 metabolite modules) (Fig. S8A and B). The correlations with *R*^2^ > 0.5 were selected (Fig. S9). In particular, CAG17 and CAG6 were positively correlated with M02 (containing tryptophan, indole, and its derivatives), M11 (including estrone 3-sulfate), and M19 (including genistein and daidzein), but negatively correlated with M28 (hormones and hormone-related compounds, including progesterone, androgen and oxidized lipids), M03 (oxidized lipids, hormones, and hormone-related compounds, e.g., oxandrolone), M18 (containing adrenosterone), and M21 (hormones and hormone-related compounds and bile acids) (Fig. S8B). We further focused on the relationships between differential bacterial species and differential hormones and hormone-related compounds. Negative correlations were observed between the abundance of Muribaculaceae, *Prevotella* spp., and *Limosilactobacillus* spp., and the concentrations of pregnenolone, allopregnan-20alpha-ol-3-one, testosterone, adrenosterone, bolasterone, and oxandrolone. In contrast, these compounds had significant positive correlations with the species from *Bifidobacterium* and *Streptococcus,* which were significantly enriched in non-return sows. This was in concordance with the findings described above, in which significant positive correlations were found between *B. pseudolongum* and progesterone-related compounds such as 5-pregnen-3β-ol-20-one and allopregnan-20alpha-ol-3-one. Furthermore, estrone 3-sulfate, daidzein, and genistein enriched in normal sows showed positive correlations with Muribaculaceae and the species from *Prevotella* and *Limosilactobacillus* but had significant negative correlations with the species from *Bifidobacterium* and *Streptococcus* (Fig. 5B and S8C).

Meanwhile, significant associations between functional pathways of the gut microbiome and fecal metabolite modules were identified (Fig. S10). Notably, steroid hormone synthesis, ovarian steroidogenesis, and the estrogen signaling pathway, which were significantly enriched in the gut microbiome of normal return sows, showed significantly positive associations with M11 (lysophosphatidyle thanolamine, lysophosphatidyl choline, indole and its derivatives, and estrogens such as estrone 3-sulfate), but were negatively correlated with M28 (hormones and hormone-related compounds, e.g., progesterone and androgen), and M03 (oxidized lipids, and hormones and hormone-related compounds including oxandrolone). Overall, the findings indicated that the changes in the gut microbiome and its functional capacities could modulate the metabolites of hormones and hormone-related compounds that affected the estrus return of weaning sows.

### Integrated analysis of the abundance of bacterial species, metagenome, and fecal metabolome confirmed the relationships between estrus return related-gut microbiome and the biosynthesis of steroid hormones

**Fig. 6:**
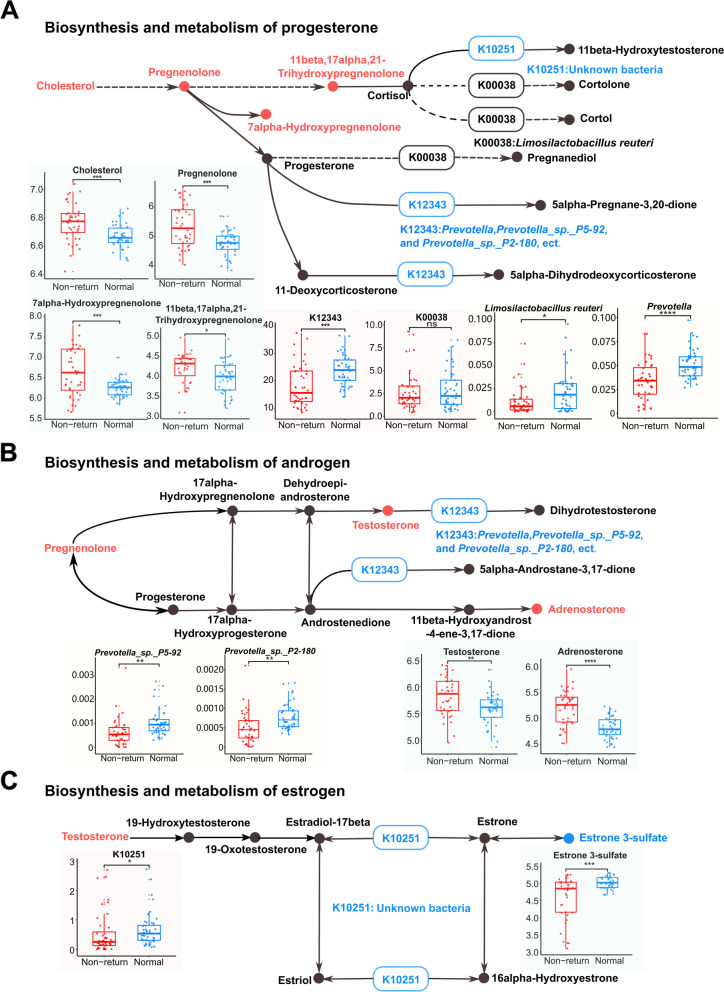
The bacterial taxa and microbial genes taking part in the biosynthesis and metabolism of sex steroid hormones and comparisons of their abundances and its metabolites between normal return and non-return sows. **A** Biosynthesis and metabolism of pregnenolone and progesterone. Microbial genes (K10251, K00038, and K12343) participating in the metabolism of progesterone were up-regulated in normal group. Moreover, *Prevotella* spp. and *Limosilactobacillus reuteri* carrying K12343 and K00038 genes also showed significant enrichment in normal group, suggesting a promotion of progesterone degradation in normal group. However, cholesterol, pregnenolone, and 11beta,17alpha,21-trihydroxypregnenolone and 7alpha-hydroxypregnenolone had higher concentrations in feces of non-return sows. **B** Biosynthesis and metabolism of testosterone. As described in **A**, microbial gene K12343 participating in the metabolism of testosterone and androstenedione, and *Prevotella* spp. carrying K12343 had higher abundances in normal sows. **C** Biosynthesis of estrogens (estradiol-17beta, estrone and estriol). Microbial gene K10251 involved in the mutual transformation of estrogens was enriched in normal sows. Boxplots show the differential abundances of bacterial species, microbial genes and fecal metabolites between non-return and normal return sows. Red represents the metabolites that were significantly enriched in non-return group, and blue represents bacterial species, microbial genes and metabolites that were significantly enriched in normal group. Wilcoxon rank sum test was used for differential analysis of bacterial species and microbial genes, and Student's* t*-test was used for differential analysis of fecal metabolites. ns, FDR > 0.05, ^*^FDR < 0.05, ^**^FDR < 0.01, ^***^FDR < 0.001, ^****^FDR < 0.0001. Gut bacterial species carrying microbial genes involved in this pathway have also been highlighted. And the solid lines between metabolites represent direct and previously confirmed pathways and the dashed lines represent indirect or unknown pathways

An integrated analysis of shotgun metagenomic sequencing and fecal metabolome data was performed to check whether the disturbance of steroid hormone biosynthesis was particularly relevant to the changes in the gut microbiome in non-return sows. Three main steps are involved in the biosynthesis of steroid hormones from cholesterol to estrogens and the reciprocal conversion between steroid hormones. (I) Biosynthesis of pregnenolone from cholesterol and its transformation and metabolism. Pregnenolone is synthesized from cholesterol. Pregnenolone and progesterone can be interconverted by a bifunctional enzyme complex. The microbial genes K10251, K12343, and K00038 are involved in the metabolism of pregnenolone and progesterone. In this study, K12343 and K10251 were significantly enriched in the gut microbiome of normal return sows. K00038 showed a similar trend, but it did not achieve a significant level. Notably, four metabolites involved in this step, including cholesterol, pregnenolone, 7alpha-hydroxypregnenolone, and 11beta, 17alpha, and 21-trihydroxypregnenolone, were detected in the fecal metabolome and had significantly lower concentrations in the feces of normal return sows. (II) Biosynthesis of testosterone from pregnenolone and progesterone, and its transformation. Pregnenolone and progesterone can be converted into 17-alpha-hydroxypregnenolone and 17-alpha-hydroxyprogesterone, respectively, and then to androstenedione. Androstenedione and testosterone can be interconverted. The microbial gene K12343 is involved in the metabolism of androstenedione and testosterone, and this had a higher abundance in normal return sows as mentioned above. From the fecal metabolome data, testosterone and adrenosterone had significantly higher abundances in non-return sows. (III) Biosynthesis of estrogens (estradiol-17beta, estrone, and estriol) and their mutual transformation. The microbial gene K10251 is involved in the process of mutual transformation of estrone and estradiol-17beta and was enriched in normal return sows. Estrone 3-sulfate, the product of the interconversion from estrone, was also enriched in normal return sows. Overall, the abundances of the microbial genes K12343, K00038, and K10251 were higher in the gut microbiome of normal return sows in both the discovery and validation sow cohorts (Fig. S11), suggesting increases in both the biosynthesis of estrogen and the degradation of progesterone and androgen in these sows. Notably, K00038 and K12343 were identified in the gut bacterial species enriched in normal return sows such as *L. reuteri* and *Prevotella* spp*.* (Fig. 1D). Moreover, strongly positive correlations were observed between K00038 and *L. reuteri* and between K12343 and *Prevotella* spp*.* (Fig. S5E). It implied that *L. reuteri* and the species from *Prevotella* could participate in the degradation of pregnenolone, progesterone, and testosterone, but promote the biosynthesis of estrogens, finally resulting in the normal return of estrus (Fig. 6).

### Biomarkers based on microbial composition and fecal metabolome for predicting non-return sows

**Fig. 7:**
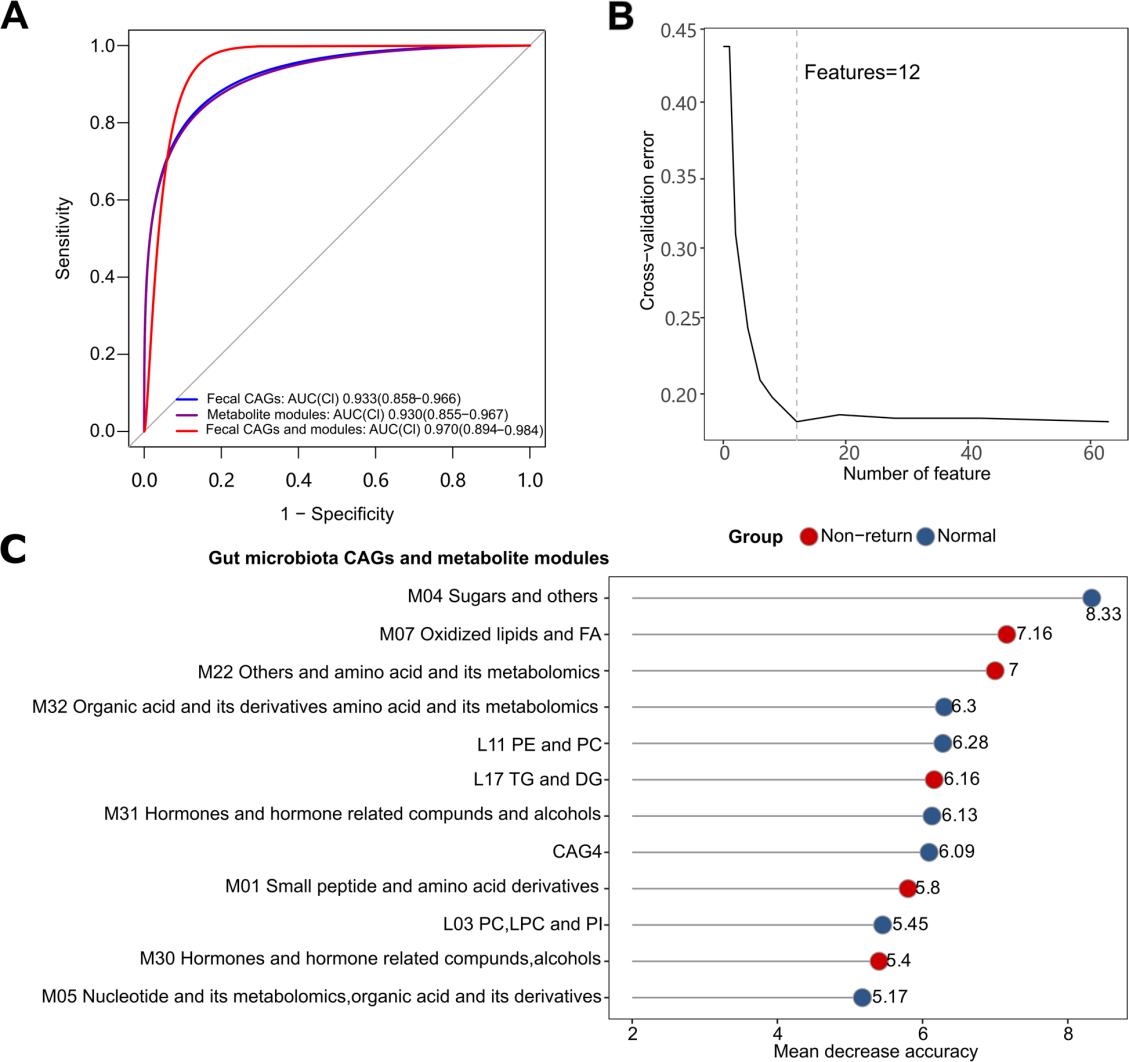
The performance of classification models based on fecal microbiota CAGs and metabolite modules in discriminating non-return sows from normal sows. **A** Aera under the receiver operating characteristic curve (AUC) of three random forest models with biomarkers of fecal microbiota CAGs and metabolite modules. The diagnostic performance was assessed using receiver operating characteristic analysis. Colors represent different models. Cl, confidence intervals. **B** Ten folds in random forest cross-validation for feature selection **C** The classification model with the best diagnostic performance is based on the gut microbiota CAGs and metabolite modules. The length of the line represents the importance of biomarkers. The red dots represent biomarkers significantly enriched in non-return sows, while blue dots represent biomarkers significantly enriched in normal sows

To evaluate whether gut microbial CAGs and metabolite modules could be used as biomarkers to discriminate non-return sows from normal sows, three random forest models were constructed based on 7 differential gut bacterial species CAGs, 56 differential metabolite modules, and the combination of differential gut microbial CAGs and metabolite modules (Fig. 7A and B). We found that single-type biomarker panels could distinguish non-return sows, with area under the curve (AUC) values of 0.933 and 0.930 (CAGs of fecal bacterial species: AUC = 0.933, metabolite modules: AUC = 0.930). The impact of discriminatory features in each type of biomarker on the predictions is shown in Fig. 7C, S12A and B. We found that the marker panels integrating fecal microbial CAGs and metabolite modules enabled the discrimination of non-return sows with the highest prediction power (AUC = 0.970).

## Discussion

The failure to return to estrus of sows after weaning has a serious influence on reproductive performance and leads to economic losses in the swine industry. There are a variety of factors affecting sow estrus return. Our previous study implied that the gut microbiota could be one of the key factors involved [14]. Few studies have investigated the effect of gut microbiota on sow estrus return after weaning. To our knowledge, there has been no study investigating the underlying mechanisms of gut microbiota influencing sow estrus. In the current study, we systemically explored the association of the gut microbiome with estrus return in 236 sows from two sow cohorts. By integrating metagenomic sequencing data, fecal metabolome data, and the genome of *P. copri* isolates, we further investigated the possible mechanism of gut microbial taxa affecting estrus return of weaning sows. Our results suggested that the changes in the gut microbiome were associated with the disturbance to the biosynthesis of host sex steroid hormones, and finally were related to the failure of sows to return to estrus.

The gut microbial composition of sows could be influenced by different parities [38]. However, in this study, we did not observe a significant effect of parity on the composition of the gut microbiota (Fig. S3). This could be because the parities of most experimental sows were relatively concentrated at the fourth to seventh parity. All experimental sows were raised on the same farm under the same conditions, with similar management practices. The non-return sows were characterized by an increased α-diversity of the gut microbiota. This was consistent with our previous study [14]. This result could be related to the levels of sex steroid hormones in sows after weaning. It has been reported that the α-diversity of the gut microbiota is inversely correlated with the level of estrogen and positively correlated with the level of testosterone [39]. However, another study showed a negative correlation between the α-diversity of the gut microbiota and the level of testosterone [40]. This may have been due to the state of reproductive physiology of the experimental animals.

We then speculated as to the possible mechanism of the gut microbiome affecting sow estrus return by integrating information concerning differential bacterial taxa, potential functional capacities, and the fecal metabolome. The species from *Muribaculaceae* [41]*, Lachnospiraceae_XPB1014_group* [42, 43], *Prevotella* [44, 45], and *Lactobacillus* [46] that can produce short-chain fatty acids (SCFAs) with anti-inflammatory effects [47] by degrading a variety of complex carbohydrates showed enrichment in the gut microbiota of normal return sows, while species from *Streptococcus*, *Enterococcus*, *Bacteroides*, and *Bifidobacterium* were enriched in non-return sows. Previous reports have suggested that the abundance of anti-inflammatory bacteria including *Lactobacillus* and *Butyrivibrio* were positively correlated with SCFAs, follicle-stimulating hormone (FSH), E2, and IL-10. Conversely, the abundances of *Actinobacteria*, *Bacteroides*, and *Streptococcus* were negatively correlated with SCFAs, FSH, E2, and IL-10 [48]. The abundance of *Bifidobacterium* spp. in the gut of women and mice was increased by increased progesterone during late pregnancy [49]. This agreed with our findings in which significantly positive correlations were found between *B. pseudolongum* and progesterone-related compounds such as 5-pregnen-3β-ol-20-one and allopregnan-20alpha-ol-3-one. From the metagenomic sequencing data obtained in this study, *Prevotella* (especially *P. copri*), *Muribaculaceae*, and *Limosilactobacillus* enriched in normal return sows harbored a β-glucosidase gene that can deconjugate estrogens in their genomes. This could increase sow estrogen levels by deconjugating estrogen and promoting its reuptake. *Corynebacterium* is not only a pathogenic bacterium [50, 51], but also harbors genes that are involved in the degradation of steroid hormones. Metagenomic analysis further showed that microbial genes having higher abundance in normal return sows were enriched in the KEGG pathways of steroid hormone biosynthesis, estrogen signaling pathway, GnRH signaling pathway, and ovarian steroidogenesis. Fecal metabolome analysis identified the relationships between the changes in the gut microbial taxa and the shifts in the fecal levels of sow sex steroid hormones and the related metabolite compounds. As is well known, progesterone, testosterone, and estrogen can be reciprocally converted. *L. reuteri* and *Prevotella* spp. harbor genes participating in the reciprocal conversion of progesterone, testosterone, and estrogen (Fig. 6). The metabolome analysis found that estrogen-related compounds, including estradiol-17phenylpropionate, estrone 3-sulfate, and phytoestrogens such as genistein and daidzein were enriched in normal sows. Estrone 3-sulfate is a long-term repository of estrone and 17β-estradiol in vivo [52]. Daidzein and genistein are isoflavones with a structure similar to estrogen, and they are also known as phytoestrogens that can modulate steroid hormone receptors and bind to estrogen receptors [53]. In this study, daidzein and genistein were positively correlated with the bacterial species enriched in normal return sows. Estrogen can also upregulate the expression of phosphatidylethanolamine N-methyltransferase, which can convert PE into phosphatidylcholine (PC) [54]. The abundance of PC is closely related to lipoprotein formation and metabolism. The impairment of lipoprotein metabolism disrupts ovarian steroid hormone synthesis [55, 56]. The changes in PC abundance during lactation in sows may indirectly impact ovarian function and steroid hormone biosynthesis, thereby affecting the post-weaning return to estrus [57]. Notably, PC had a higher abundance in normal return sows and was positively correlated with normal return-associated bacterial species. Testosterone and other androgen-related compounds, including oxandrolone, adrenosterone, bolasterone, and testosterone cypionate, had higher abundances in non-return sows. Androgen has an antagonistic effect on estrogen, and excessive accumulation of androgen can interfere with the normal development of follicles [58]. Progesterone-related compounds such as pregnenolone and allopregnan-20alpha-ol-3-one also showed enrichment in non-return sows. An increased level of progesterone has a negative feedback on the hypothalamus and pituitary gland, inhibiting FSH secretion and preventing follicle development [59]. Overall, we inferred that the shifts in the concentrations of sex hormones and hormone-related compounds caused by the changes in the gut microbiome could be one of the reasons leading to the failure of sows to return to estrus.

Sugars, vitamin E, and the lipid molecules triglyceride and diacylglycerol had higher abundances in normal return sows and were negatively correlated with non-return-associated bacterial species. An adequate supply of energy and nutrients is required in sows after weaning due to the loss of energy and body weight during lactation. During sow lactation, sugars, vitamins, triglyceride, and diacylglycerol should be supplied to compensate for the energy loss and to meet the nutritional requirements [60, 61]. A deficiency of vitamin E inhibits the regulation of the pituitary gland in the secretion of estrogen in the ovaries, and this can induce menopause syndrome and premature ovarian failure [62]. Phosphate sugars and lipid molecule ceramides were enriched in non-return sows and were positively correlated with non-return-associated bacterial species. The accumulation of phosphorylated sugars during cellular metabolism has wide-ranging toxic effects on organisms [63]. Ceramides are metabolites that accumulate in individuals with obesity or dyslipidemia [64], and the inhibition of ceramide synthesis ameliorates glucocorticoid-, saturated-fat-, and obesity-induced insulin resistance [65]. Insulin resistance and compensatory hyperinsulinemia can induce reproductive diseases related to excessive androgen levels such as PCOS that shows abnormal estrous cycles, as insulin can induce androgen secretion from the adrenal glands and regulate the level of luteinizing hormone [66, 67]. Notably, the metabolites having higher abundance in normal return sows were enriched in the pathway of valine, leucine, and isoleucine degradation. It has been reported that increased levels of branched-chain amino acids are related to insulin resistance and obesity [16]. Obesity can perturb the processes related to female fertility, including sex hormone secretion [68]. The compounds enriched in normal sows were also related to tryptophan metabolism. Gut microbes, e.g., *Lactobacillus*, can convert tryptophan into indole and its derivatives. *Limosilactobacillus* and *Lactobacillus* spp. were positively correlated with fecal concentrations of tryptophan as well as indole and its derivatives. Indole and its derivatives, including indole-3-acid-acetic and indole acetaldehyde, were also enriched in normal sows; these can act as ligands for the aryl hydrocarbon receptor [69] that plays an important role in intestinal barrier function and intestinal homeostasis [70]. These results suggested that some metabolites produced by gut microbiota should be provided as energy and nutrients to sows and that they may cause sow metabolic disorders, e.g., obesity. This should be another factor influencing sow estrus return.

Identifying the specific biomarkers of the gut microbiota and fecal metabolites that can serve as predictive indicators of post-weaning estrus performance in sows are economically important for the pig industry. Such information can help to identify sows with a shortened weaning-to-estrus interval, which can significantly improve sow reproductive efficiency and production performance. In this study, the biomarkers combining both gut microbiota and fecal metabolome could be used to predict non-return sows with high accuracy. We also suggested that *L. reuteri* and *Prevotella* spp. should be potential candidate probiotics for regulating estrus return of weaning sows, although the causality and the underlying mechanisms need to be confirmed. The results also suggested that in addition to genetic improvement, nutrition, and management, it is important to focus on the regulation of gut microbial compositions of sows in breeding programs.

## Conclusions

In this study, we found that the composition of the gut microbiome was significantly associated with sow estrus return after weaning. The shifts in the concentrations of sex hormones and hormone-related compounds caused by the changes in gut microbiome could be an important factor influencing sow estrus return. Specifically, *L. reuteri* and *Prevotella* spp. may regulate sow estrus return by participating in the reciprocal conversion of pregnenolone, progesterone, testosterone, and estrogen. The metabolites that were produced by gut microbiota should be provided as energy and nutrients to sows, as their lack may cause sow metabolic disorders, a factor possibly influencing sow estrus return. Although the causality and the underlying mechanisms should need to be further confirmed using various experiments, we have provided valuable evidence for the hypothesis that the manipulation of gut microbiota may be an effective way to improve the estrus return of sows after weaning.

### Supplementary Information

**Additional file 1: Fig. S1.** Overview of the workflow for this study. **Fig. S2.** The rarefaction curve and taxonomic composition of gut microbiota in 207 fecal samples. **A** The rarefaction curve of ACE, Chao1, and Observed species index. Colors indicate grouping. **B** The Sankey diagram depicts the bacterial composition of fecal samples from experimental sows. The colored columns from left to right represent taxonomy from phylum to genus level, and the length of bar indicates the relative abundances of gut bacterial taxa. **Fig. S3.** Comparison of the microbial compositions of gut among different parities by PCoA based on Bray-Curtis distance. **Fig. S4.** Comparison of the diversity of gut microbial composition and identification of differential gut bacterial taxa between normal and non-return groups in 207 weaned sows. **A** Comparison of the alpha-diversity index of gut microbiota. **B** Principal coordinate analysis (PCoA) based on the Bray-Curtis distance shows different microbial compositions between normal return and non-return sows. **C** Boxplots of the Bray-Curtis dissimilarity of gut microbiome between subjects within and between each group. The comparison was performed by Wilcoxon rank-sum test at the significance level of *P* < 0.05. **D** and **E** Identification of the differential bacterial genera and ASVs between normal return and non-return sows at the thresholds of LDA score > 3 and FDR < 0.05. The relative abundance and the LDA score of differential bacterial taxa are shown in boxplots on the left and dots on the right, respectively. **Fig. S5.** The shifts in the gut microbiome between normal return and non-return sows with metagenomic sequencing data. **A** Comparison of gene richness between normal return and non-return sows. **B** Comparison of the abundance of *P. copri* isolate between normal return and non-return sows. Wilcoxon rank-sum test was performed. **C** Shifts in 16 genera of gut microbiota with the highest abundance between normal return and non-return sows in 85 fecal samples with shotgun metagenomic sequencing data. **D** Butterfly plot showing the differential gut bacterial taxa between two sow groups and the threshold of LDA score > 2 and *P* < 0.05. **E** The heatmap shows the Spearman’s rank correlations of KO genes involved in estrogen metabolism and steroid hormone biosynthesis with specific gut microbes that carry these KO genes. Benjamini-Hochberg adjusted *P* values, and only significant correlations are noted. ns, FDR > 0.05; *, FDR < 0.05; **, FDR < 0.01, and ***, FDR < 0.001. **Fig. S6.** Comparison of the diversity of gut microbiota and identification of gut bacterial species showing differential abundances between normal and non-return sows in the validation cohort. **A** Comparison of the alpha-diversity of gut microbiota. **B** Comparison of the gut microbial composition between normal return and non-return sows by principal coordinate analysis (PCoA) based on the Bray-Curtis distance. **C** Boxplot of the Bray-Curtis dissimilarity of gut microbiota between subjects within and between each group. Comparison was performed by Wilcoxon rank-sum test at the significance level of *P* < 0.05. **D** Differential bacterial species between normal return and non-return sows with metagenomic sequencing data. The significance threshold was set at LDA score > 2 and *P* < 0.1. **Fig. S7.** Co-abundance groups (CAGs) of bacterial species showing different abundances between normal and non-return sows and comparing the abundances of bacterial genes participating in the deconjugation of estrogen in the validation cohort. **A** The network diagram of six co-abundance groups (CAGs) shows different enrichments between normal return and non-return sows. Lines were drawn only when its correlation coefficient was greater than 0.4 and *P* < 0.05. Further details regarding the network description are provided in Fig. 2. The bacterial species in CAG2 are listed in Table S7. **B** Boxplots show the differential abundances of seven CAGs between two sow groups. Wilcoxon rank-sum test was performed for comparison analysis. *, *P* < 0.05, **, *P* < 0.01, and ***, *P* < 0.001. **C** Comparison of gene richness between normal return and non-return sows. **D** The comparisons of the abundances of β-glucuronidase, β-glucosidase and hydroxysteroid dehydrogenases that participate in the deconjugation of estrogen. Wilcoxon rank-sum test was used for the comparison, ns, FDR > 0.05. **Fig. S8.** Spearman correlation analysis between gut microbiota and fecal metabolites. **A**-**B** The heatmaps show the associations between differential CAGs and metabolic modules. **A** Lipid molecule modules. **B** Metabolite modules. **C** The heatmap shows the relationships between differential bacterial taxa, and hormones and hormone-related compounds. Spearman correlation coefficients were calculated. Red represents positive correlations and blue represents negative correlations. The stars in the grid represent the significance threshold: +, FDR < 0.05; *, FDR < 0.01; and **, FDR < 0.001. **Fig. S9.** Sankey diagram demonstrating the association between differential gut microbiota CAGs and differential fecal metabolite modules. Only those associations with Spearman correlation coefficient greater than 0.5 and FDR less than 0.05 were displayed. The weight of correlation coefficient is represented by the thickness of the connections between fecal microbiota and metabolic modules. Positive correlations were colored red, whereas negative correlations are represented by blue. In the gut microbiota column, the green stratum represents the CAGs that were significantly enriched in normal sows, the purple stratums represent the CAGs that were significantly depleted in normal sows. While in the metabolome column, the orange stratums represent metabolite modules that were significantly enriched in normal sows, and the pink stratums represent metabolite modules significantly depleted in normal sows. **Fig. S10.** The Spearman correlation analysis between differential functional capacities of gut microbiome and fecal metabolite modules. The black text on the left indicates KEGG Pathways at the level 3, and the purple text on the left indicates KEGG Pathways at the level 2. Red represents positive correlations and blue represents negative correlations. The stars in the grid indicate the significance threshold: +, FDR < 0.05; *, FDR < 0.01, and **, FDR < 0.001. **Fig. S11.** Comparing the abundances of microbial genes involved in the biosynthesis and metabolism of sex steroid hormones between normal return and non-return sows in the validation cohort. **Fig. S12.** Biomarkers based on gut microbial CAGs and metabolite modules for discriminating non-return from normal return sows. A Biomarkers of gut microbiota CAGs. B Biomarkers of fecal metabolite modules. The length of the lines indicates the importance of biomarkers. The red dots represent the biomarkers significantly enriched in non-return sows, while blue dots represent the biomarkers significantly enriched in normal sows.

**Additional file 2: Table S1.** Nutrient components of commercial formula feed provided to experimental pigs. **Table S2.** Differential ASVs and genera between normal return and non-return sows in 207 fecal samples. **Table S3.** Description of metagenomic sequencing data in 85 fecal samples from discovery cohort and 29 fecal samples from validation cohort. **Table S4.** Bacterial species and taxa showing different enrichments between normal return and non-return sows in 85 fecal samples with metagenomic sequencing data. **Table S5.** Multivariate logistic regression analysis for the relationship between gut microbial species and estrus return in experimental sows. **Table S6.** 540 species of gut microbiota used to construct co-abundance groups (CAGs) in 85 samples. **Table S7.** 495 species of gut microbiota used to construct co-abundance groups (CAGs) in validation cohort. **Table S8.** Differential CAGs of gut microbiota between normal return and non-return sows in discovery and validation cohorts. **Table S9.** Differential functional capacities of gut microbiota between normal return and non-return sows in discovery cohort. **Table S10.** Differential functional capacities of gut microbiota between normal return and non-return sows in validation cohort. **Table S11.** Differential metabolites and lipid molecules between normal return and non-return sows in 85 samples. **Table S12.** List of fecal metabolites and lipid molecules clustered into each co-abundance module (metabolite and lipid modules). **Table S13.** Differential metabolite and lipid molecule modules between normal return and non-return sows.

## Data Availability

The 16S rRNA gene sequencing data and metagenomic sequencing data were submitted to the GSA database under accession numbers: CRA008694 and CRA008698, respectively.

## Abbreviations

ASVs: Amplicon sequence variants
AUC: Area under the ROC curve
CAGs: Co-abundance groups
CUR: Curtain gas
CV: Coefficient of variation
FDR: False discovery rate
FSH: Follicle-stimulating hormone
GnRH: Gonadotropin-releasing hormone
HSD: Hydroxysteroid dehydrogenases
KEGG: Kyoto Encyclopedia of Genes and Genomes
KO genes: KEGG orthology genes
LDA: Linear discriminant analysis
LEfSe: Linear discriminate analysis effect size
LIT: Linear ion trap
MRM: Multiple reaction monitoring
OPLS-DA: Orthogonal partial least squares discriminant analysis
PC: Phosphatidylcholine
PCoA: Principal coordinate analysis
PCOS: Polycystic ovary syndrome
QC: Quality control
QQQ: Triple quadrupole
QTRAP: Triple quadrupole-linear ion trap mass spectrometer
ROC: Receiver operating characteristic
SCFAs: Short-chain fatty acids
SparCC: Sparse correlations for compositional data
VIP: Variable importance in the projection
