# Supplementary material for: Gut microbiota affects the estrus return of sows by regulating the metabolism of sex steroid hormones
Source: J Anim Sci Biotechnol. 2023 Dec 20;14:155. doi: 10.1186/s40104-023-00959-5 (PMC10731813; doi:10.1186/s40104-023-00959-5)
Supplement: Supplementary file 1 — Additional file 1: Fig. S1. Overview of the workflow for this study. Fig. S2. The rarefaction curve and taxonomic composition of gut microbiota in 207 fecal samples. A The rarefaction curve of ACE, Chao1, and Observed species index. Colors indicate grouping. B The Sankey diagram depicts the bacterial composition of fecal samples from experimental sows. The colored columns from left to right represent taxonomy from phylum to genus level, and the length of bar indicates the relative abundances of gut bacterial taxa. Fig. S3. Comparison of the microbial compositions of gut among different parities by PCoA based on Bray-Curtis distance. Fig. S4. Comparison of the diversity of gut microbial composition and identification of differential gut bacterial taxa between normal and non-return groups in 207 weaned sows. A Comparison of the alpha-diversity index of gut microbiota. B Principal coordinate analysis (PCoA) based on the Bray-Curtis distance shows different microbial compositions between normal return and non-return sows. C Boxplots of the Bray-Curtis dissimilarity of gut microbiome between subjects within and between each group. The comparison was performed by Wilcoxon rank-sum test at the significance level of P < 0.05. D and E Identification of the differential bacterial genera and ASVs between normal return and non-return sows at the thresholds of LDA score > 3 and FDR < 0.05. The relative abundance and the LDA score of differential bacterial taxa are shown in boxplots on the left and dots on the right, respectively. Fig. S5. The shifts in the gut microbiome between normal return and non-return sows with metagenomic sequencing data. A Comparison of gene richness between normal return and non-return sows. B Comparison of the abundance of P. copri isolate between normal return and non-return sows. Wilcoxon rank-sum test was performed. C Shifts in 16 genera of gut microbiota with the highest abundance between normal return and non-return sows in 85 fecal samples with s [file 40104_2023_959_MOESM1_ESM.docx]

**Additional file 1**


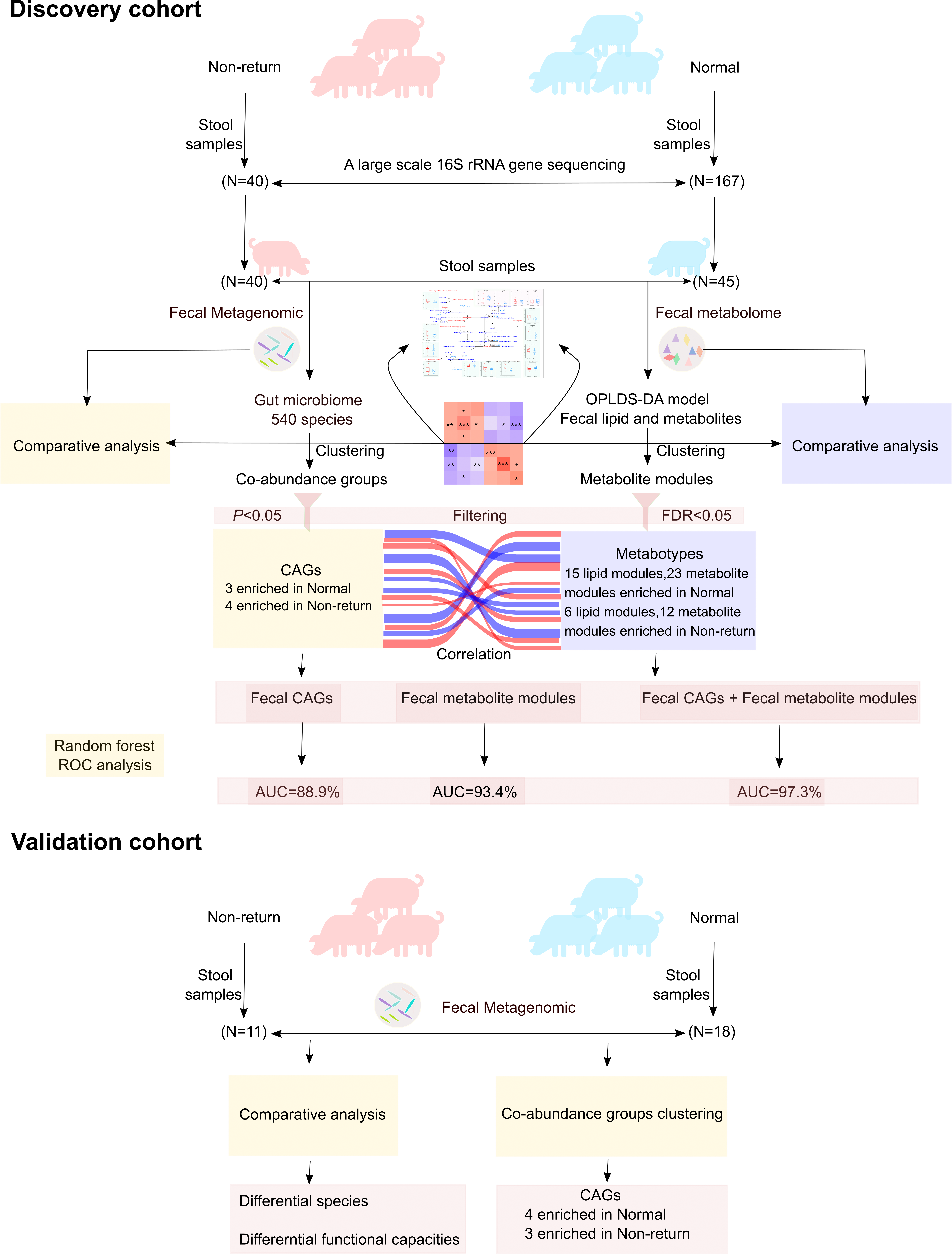


**Fig. S1** Overview of the workflow for this study

**
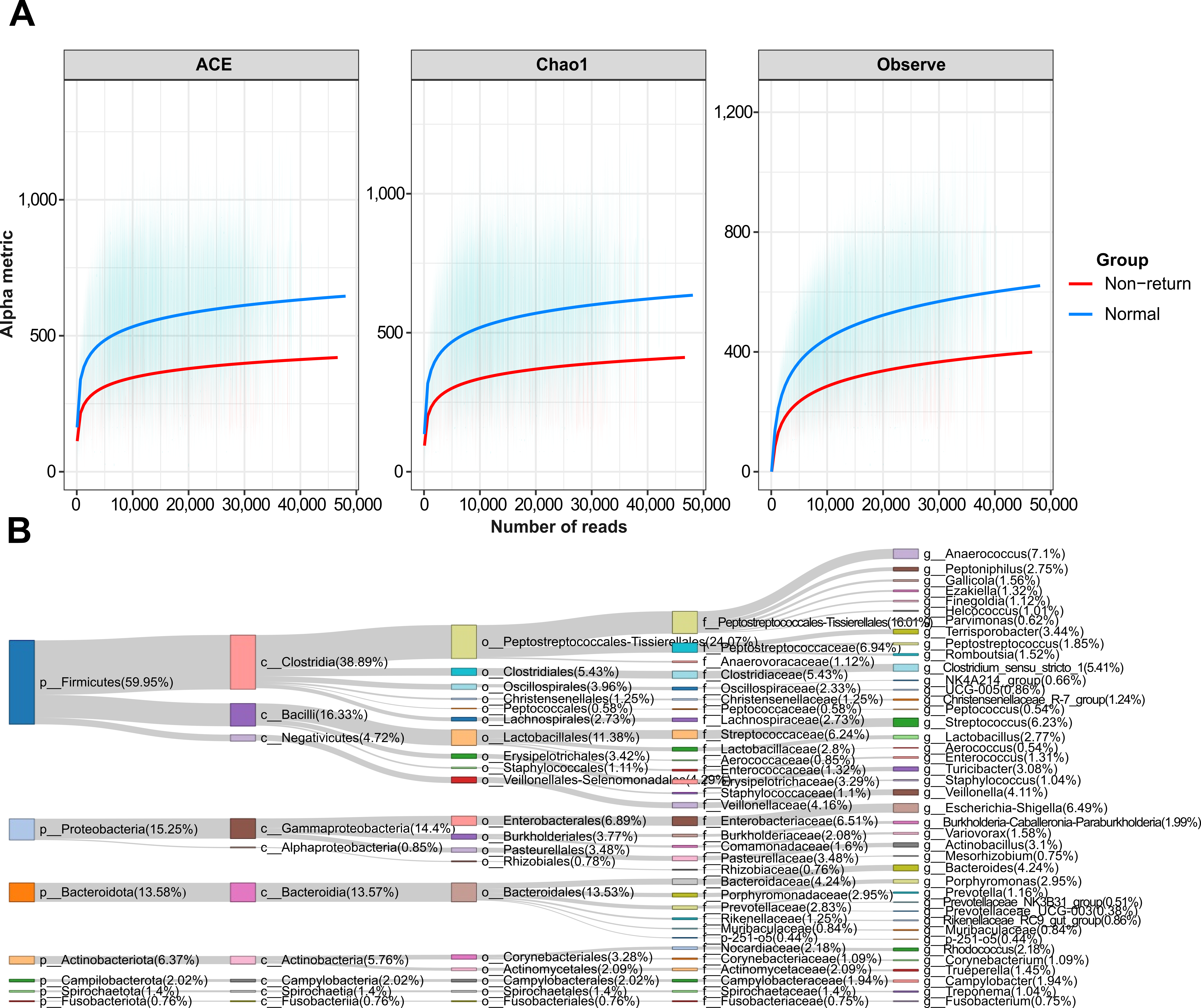
**

**Fig. S2** The rarefaction curve and taxonomic composition of gut microbiota in 207 fecal samples. **A** The rarefaction curve of ACE, Chao1, and Observed species index. Colors indicate grouping. **B** The Sankey diagram depicts the bacterial composition of fecal samples from experimental sows. The colored columns from left to right represent taxonomy from phylum to genus level, and the length of bar indicates the relative abundances of gut bacterial taxa


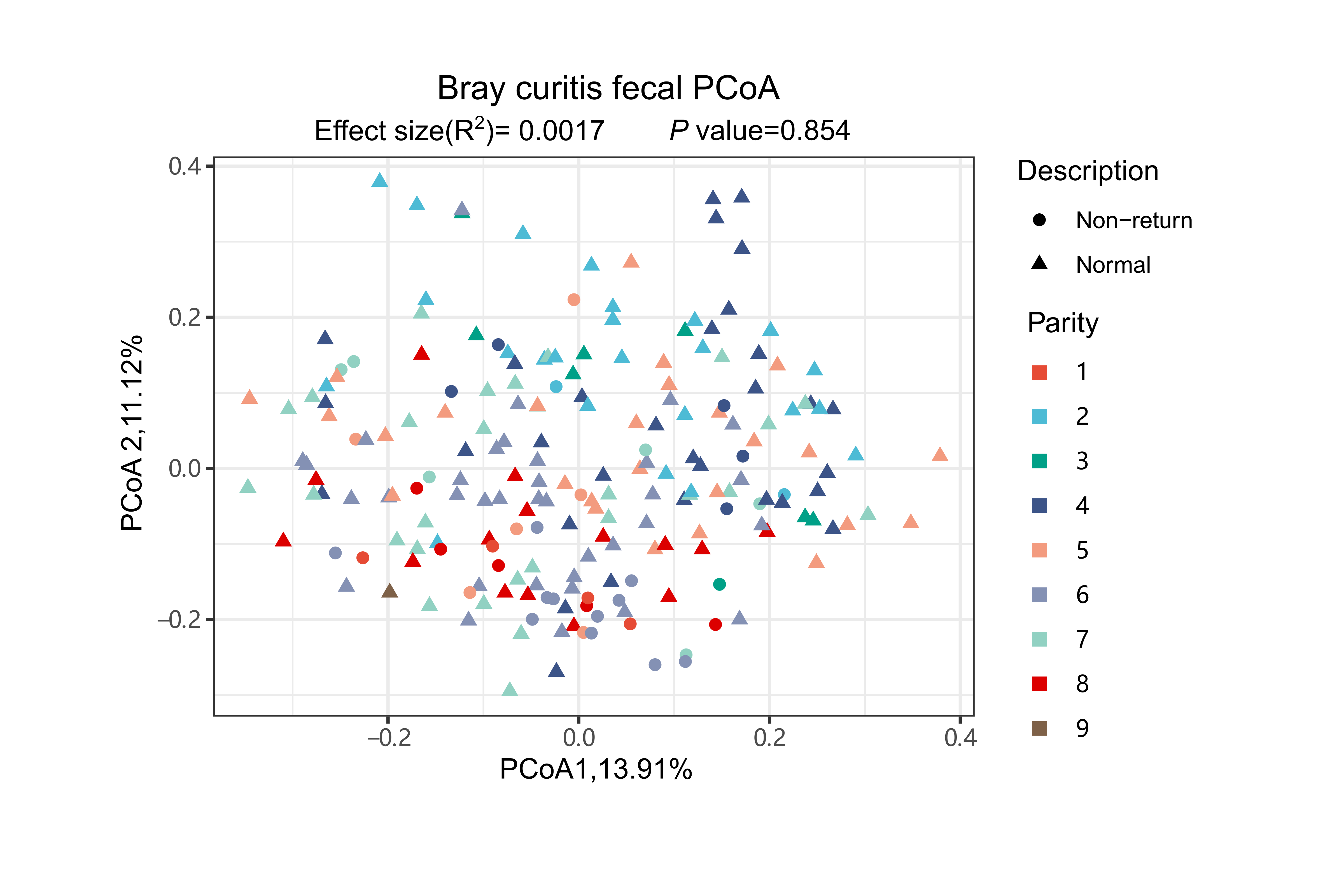


**Fig. S3** Comparison of the microbial compositions of gut among different parities by PCoA based on Bray-Curtis distance


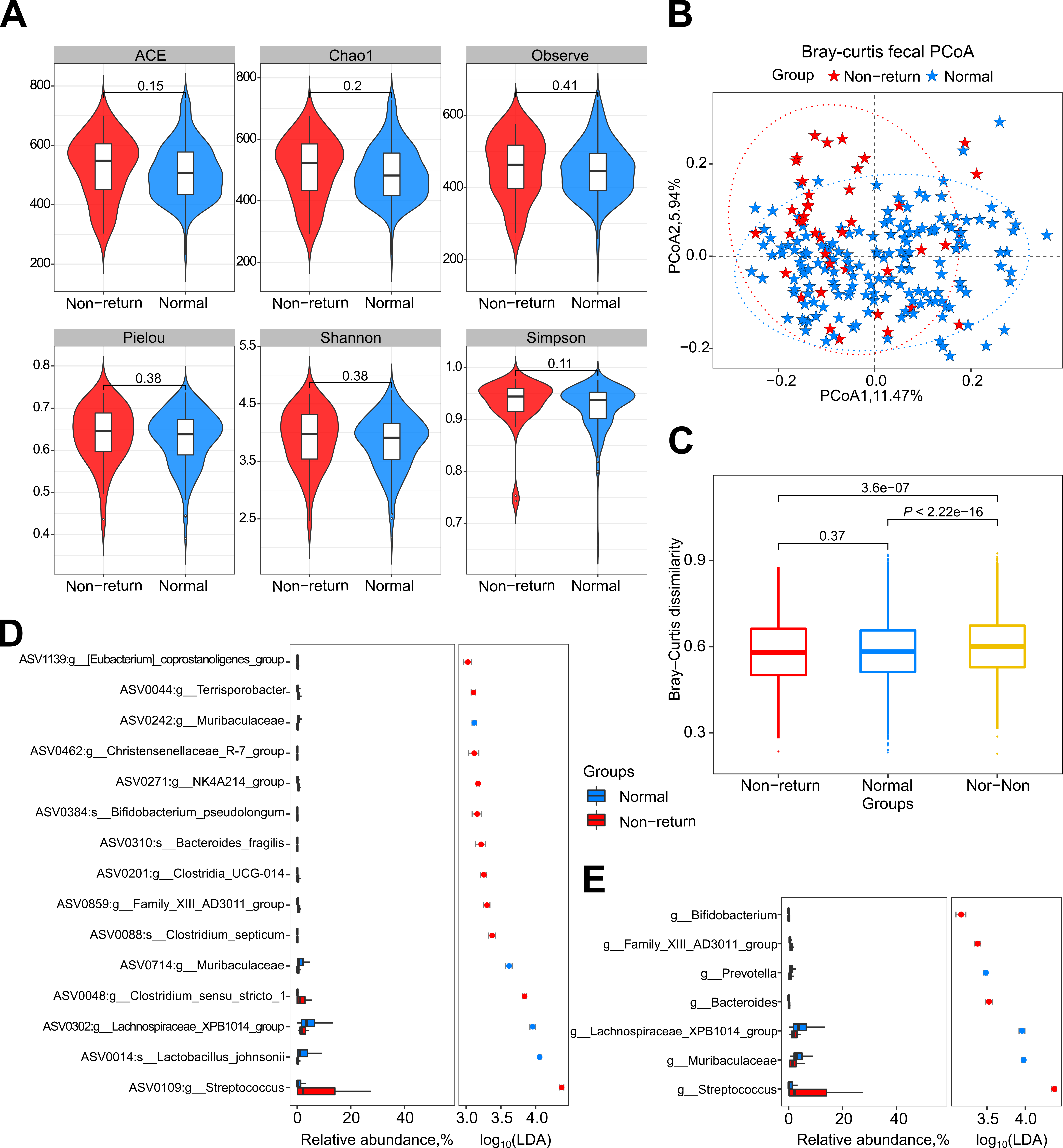


**Fig. S4** Comparison of the diversity of gut microbial composition and identification of differential gut bacterial taxa between normal and non-return groups in 207 weaned sows. **A** Comparison of the alpha-diversity index of gut microbiota. **B** Principal coordinate analysis (PCoA) based on the Bray-Curtis distance shows different microbial compositions between normal return and non-return sows. **C** Boxplots of the Bray-Curtis dissimilarity of gut microbiome between subjects within and between each group. The comparison was performed by Wilcoxon rank-sum test at the significance level of *P* < 0.05. **D** and **E** Identification of the differential bacterial genera and ASVs between normal return and non-return sows at the thresholds of LDA score > 3 and FDR < 0.05. The relative abundance and the LDA score of differential bacterial taxa are shown in boxplots on the left and dots on the right, respectively


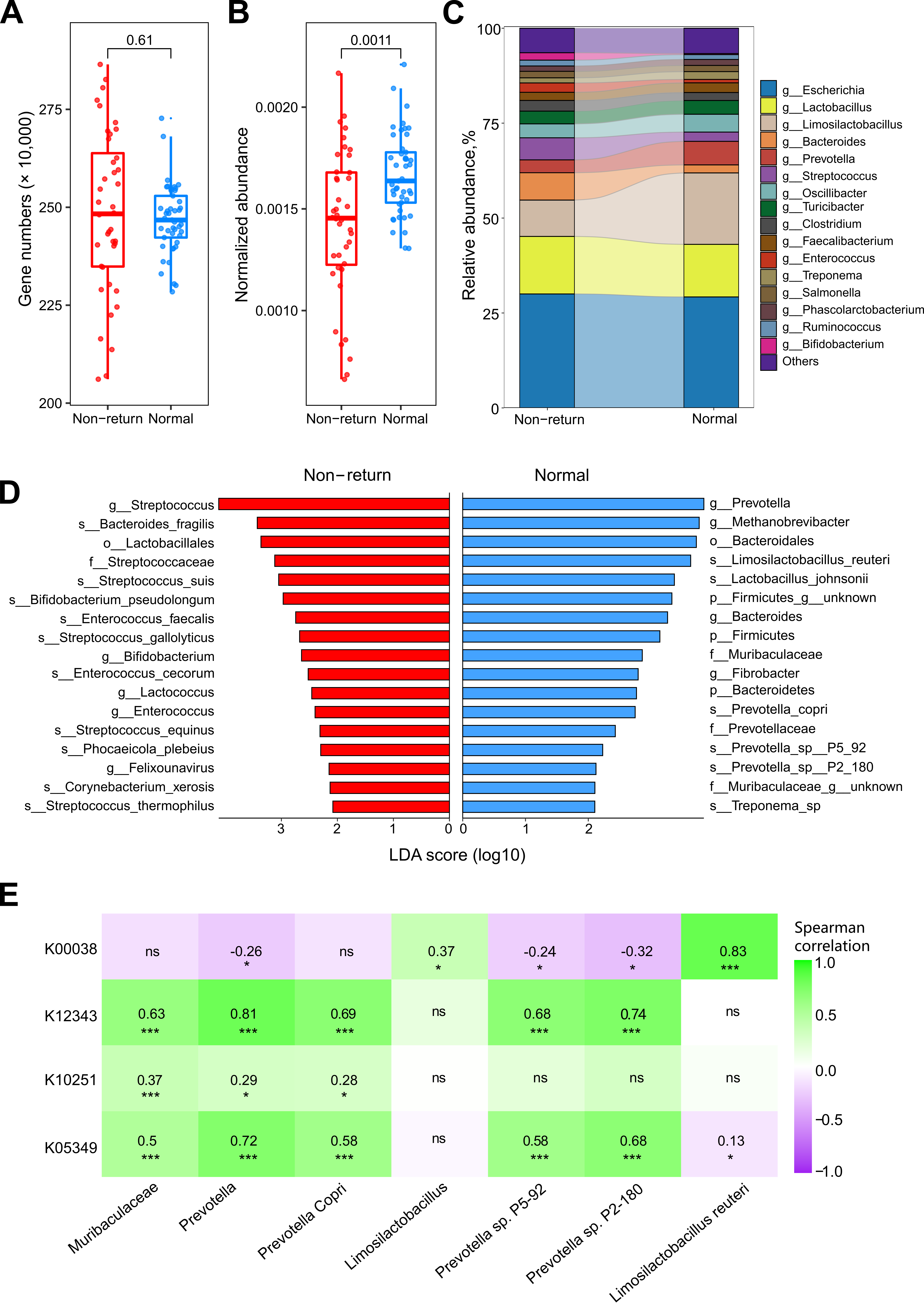


**Fig. S5** The shifts in the gut microbiome between normal return and non-return sows with metagenomic sequencing data. **A** Comparison of gene richness between normal return and non-return sows. **B** Comparison of the abundance of *P. copri* isolate between normal return and non-return sows. Wilcoxon rank-sum test was performed. **C** Shifts in 16 genera of gut microbiota with the highest abundance between normal return and non-return sows in 85 fecal samples with shotgun metagenomic sequencing data. **D** Butterfly plot showing the differential gut bacterial taxa between two sow groups and the threshold of LDA score > 2 and *P* < 0.05. **E** The heatmap shows the Spearman’s rank correlations of KO genes involved in estrogen metabolism and steroid hormone biosynthesis with specific gut microbes that carry these KO genes. Benjamini-Hochberg adjusted *P* values, and only significant correlations are noted. ns, FDR > 0.05; *, FDR < 0.05; **, FDR < 0.01, and ***, FDR < 0.001


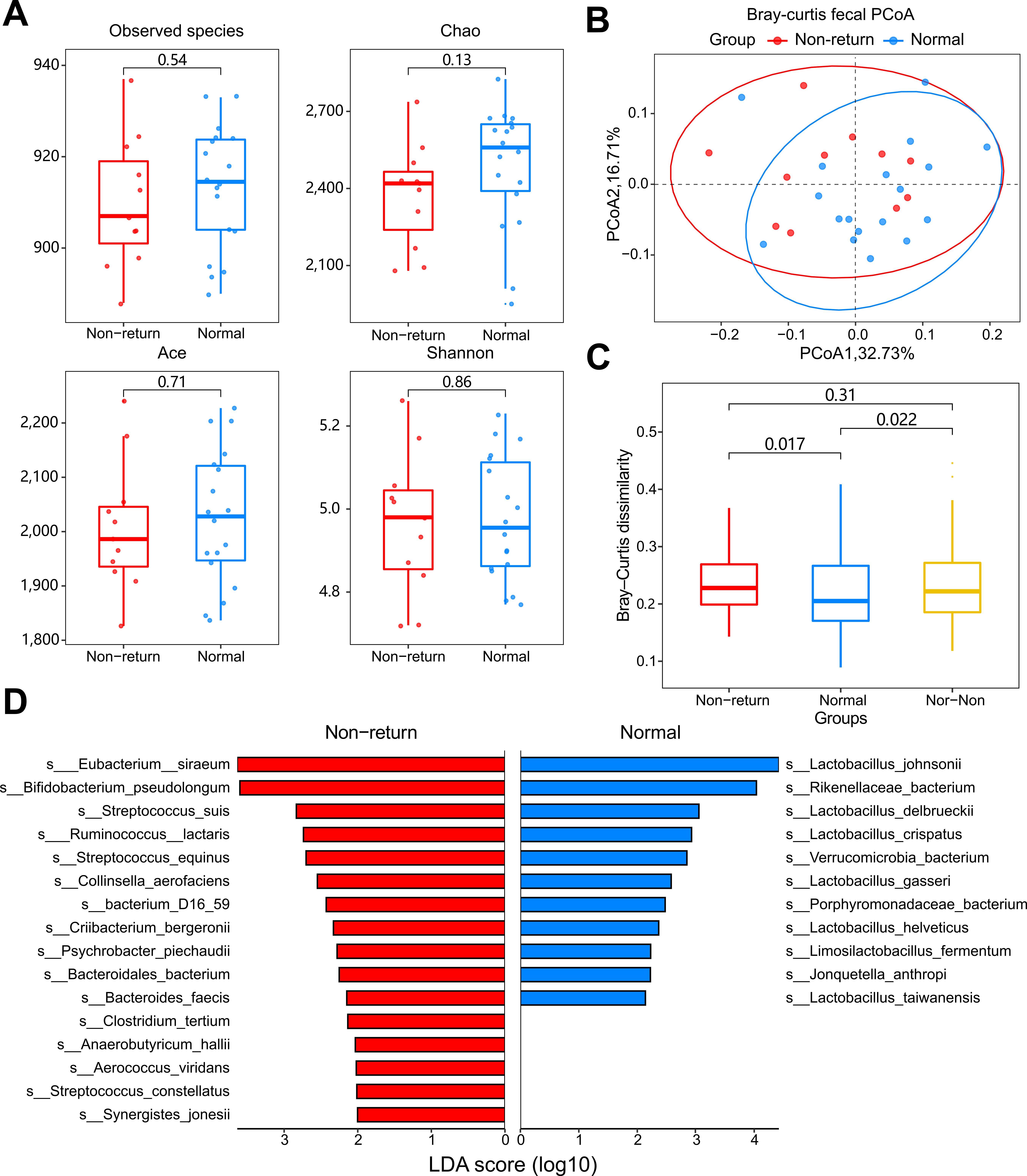


**Fig. S6** Comparison of the diversity of gut microbiota and identification of gut bacterial species showing differential abundances between normal and non-return sows in the validation cohort. **A** Comparison of the alpha-diversity of gut microbiota. **B** Comparison of the gut microbial composition between normal return and non-return sows by principal coordinate analysis (PCoA) based on the Bray-Curtis distance. **C** Boxplot of the Bray-Curtis dissimilarity of gut microbiota between subjects within and between each group. Comparison was performed by Wilcoxon rank-sum test at the significance level of *P* < 0.05. **D** Differential bacterial species between normal return and non-return sows with metagenomic sequencing data. The significance threshold was set at LDA score > 2 and *P* < 0.1


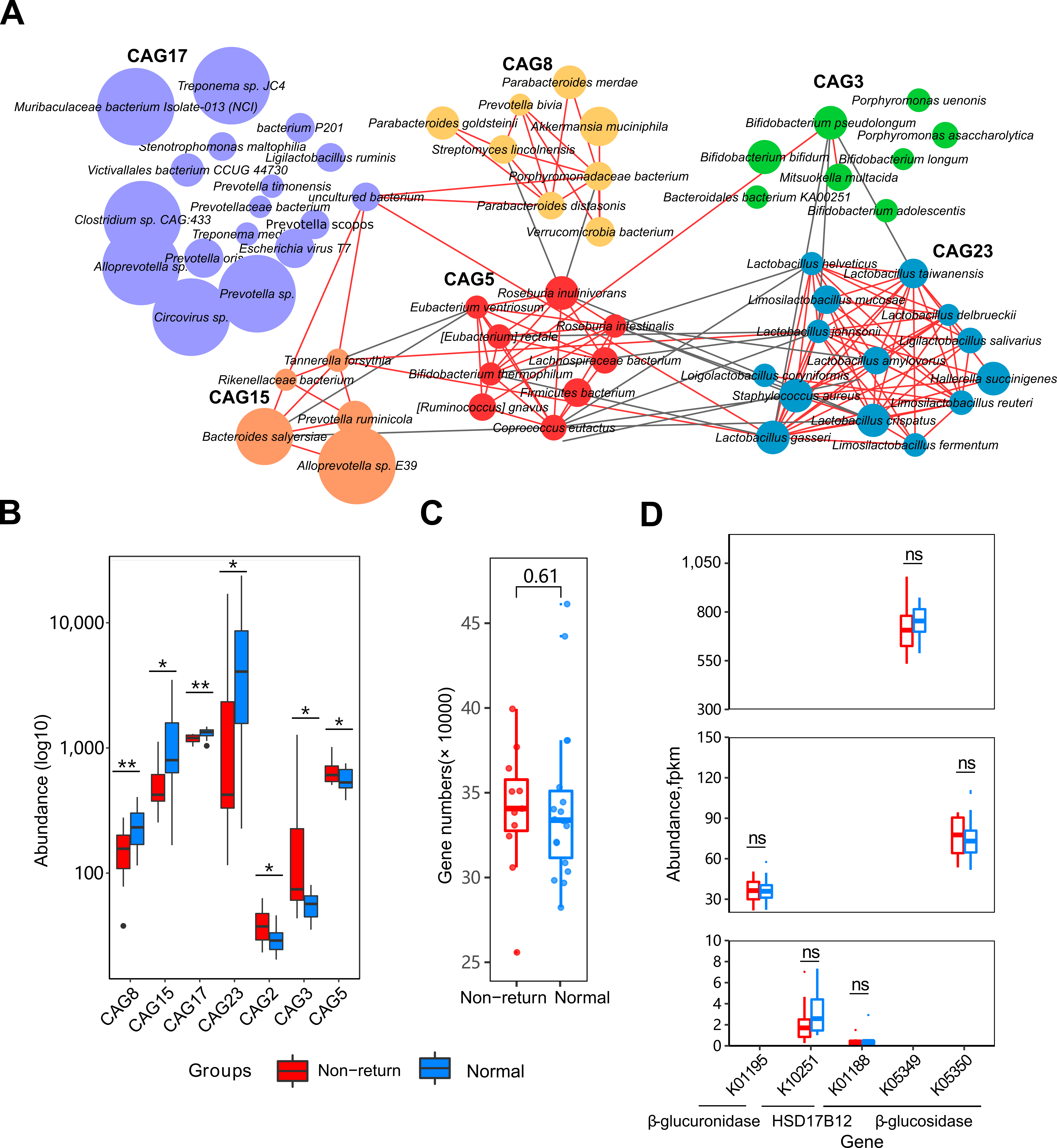


**Fig. S7** Co-abundance groups (CAGs) of bacterial species showing different abundances between normal and non-return sows and comparing the abundances of bacterial genes participating in the deconjugation of estrogen in the validation cohort. **A** The network diagram of six co-abundance groups (CAGs) shows different enrichments between normal return and non-return sows. Lines were drawn only when its correlation coefficient was greater than 0.4 and *P* < 0.05. Further details regarding the network description are provided in Fig. 2. The bacterial species in CAG2 are listed in Table S7. **B** Boxplots show the differential abundances of seven CAGs between two sow groups. Wilcoxon rank-sum test was performed for comparison analysis. *, *P* < 0.05, **, *P* < 0.01, and ***, *P* < 0.001. **C** Comparison of gene richness between normal return and non-return sows. **D** The comparisons of the abundances of β-glucuronidase, β-glucosidase and hydroxysteroid dehydrogenases that participate in the deconjugation of estrogen. Wilcoxon rank-sum test was used for the comparison, ns, FDR > 0.05


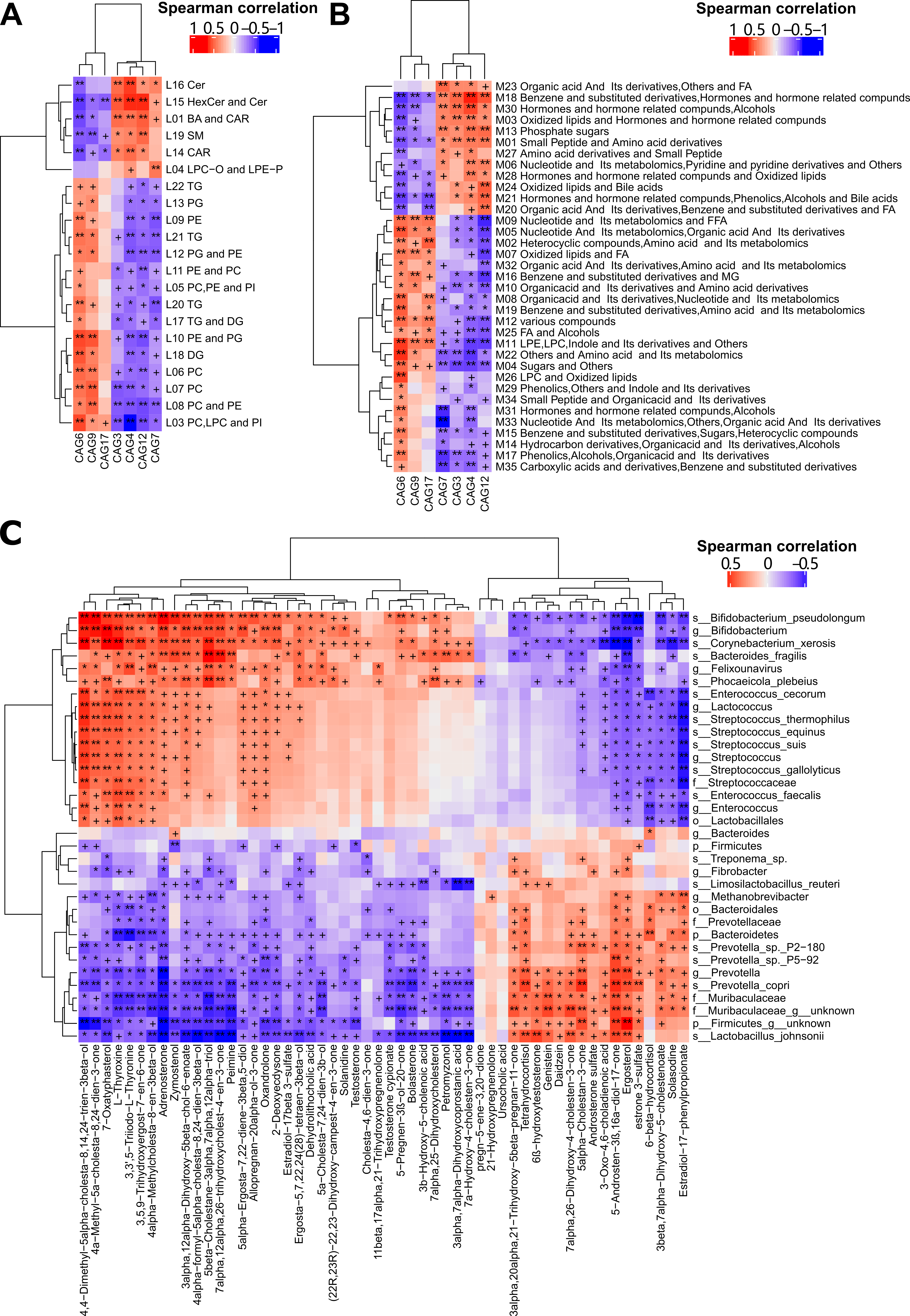


**Fig. S8** Spearman correlation analysis between gut microbiota and fecal metabolites. **A-B** The heatmaps show the associations between differential CAGs and metabolic modules. **A** Lipid molecule modules. **B** Metabolite modules. **C** The heatmap shows the relationships between differential bacterial taxa, and hormones and hormone-related compounds. Spearman correlation coefficients were calculated. Red represents positive correlations and blue represents negative correlations. The stars in the grid represent the significance threshold: +, FDR < 0.05; *, FDR < 0.01; and **, FDR < 0.001


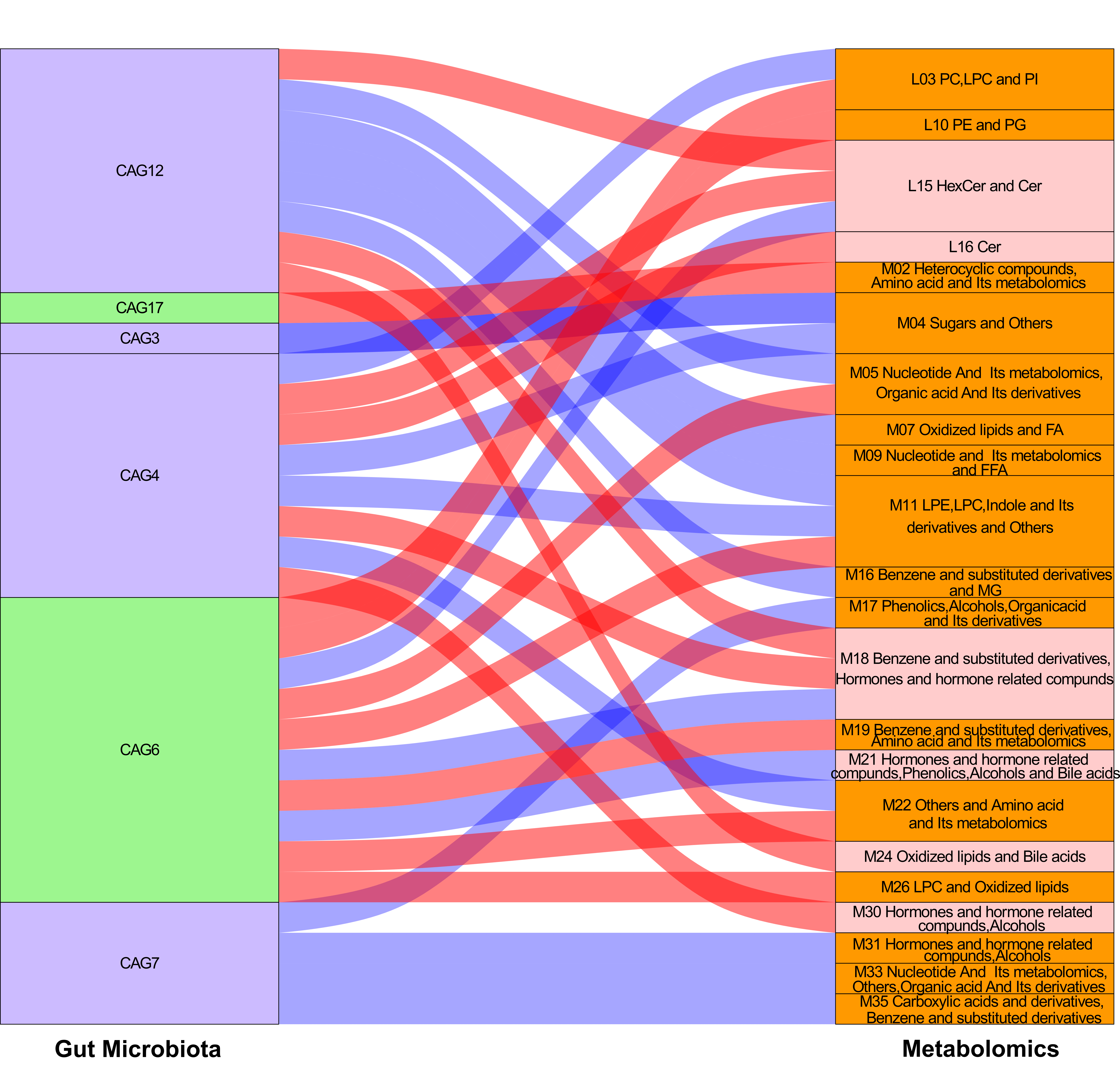


**Fig. S9** Sankey diagram demonstrating the association between differential gut microbiota CAGs and differential fecal metabolite modules. Only those associations with Spearman correlation coefficient greater than 0.5 and FDR less than 0.05 were displayed. The weight of correlation coefficient is represented by the thickness of the connections between fecal microbiota and metabolic modules. Positive correlations were colored red, whereas negative correlations are represented by blue. In the gut microbiota column, the green stratum represents the CAGs that were significantly enriched in normal sows, the purple stratums represent the CAGs that were significantly depleted in normal sows. While in the metabolome column, the orange stratums represent metabolite modules that were significantly enriched in normal sows, and the pink stratums represent metabolite modules significantly depleted in normal sows


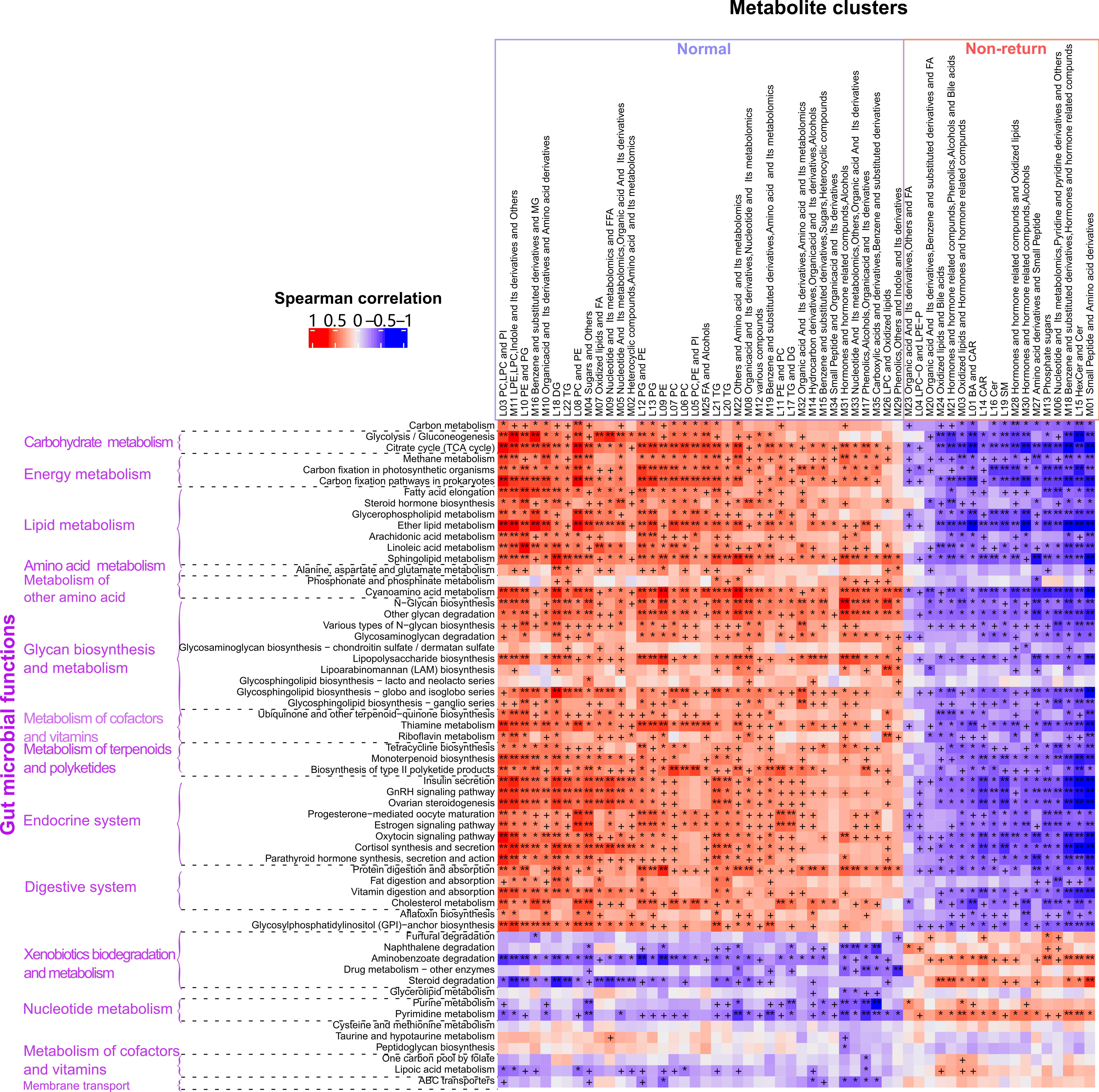


**Fig. S10** The Spearman correlation analysis between differential functional capacities of gut microbiome and fecal metabolite modules. The black text on the left indicates KEGG Pathways at the level 3, and the purple text on the left indicates KEGG Pathways at the level 2. Red represents positive correlations and blue represents negative correlations. The stars in the grid indicate the significance threshold: +, FDR < 0.05; *, FDR < 0.01, and **, FDR < 0.001


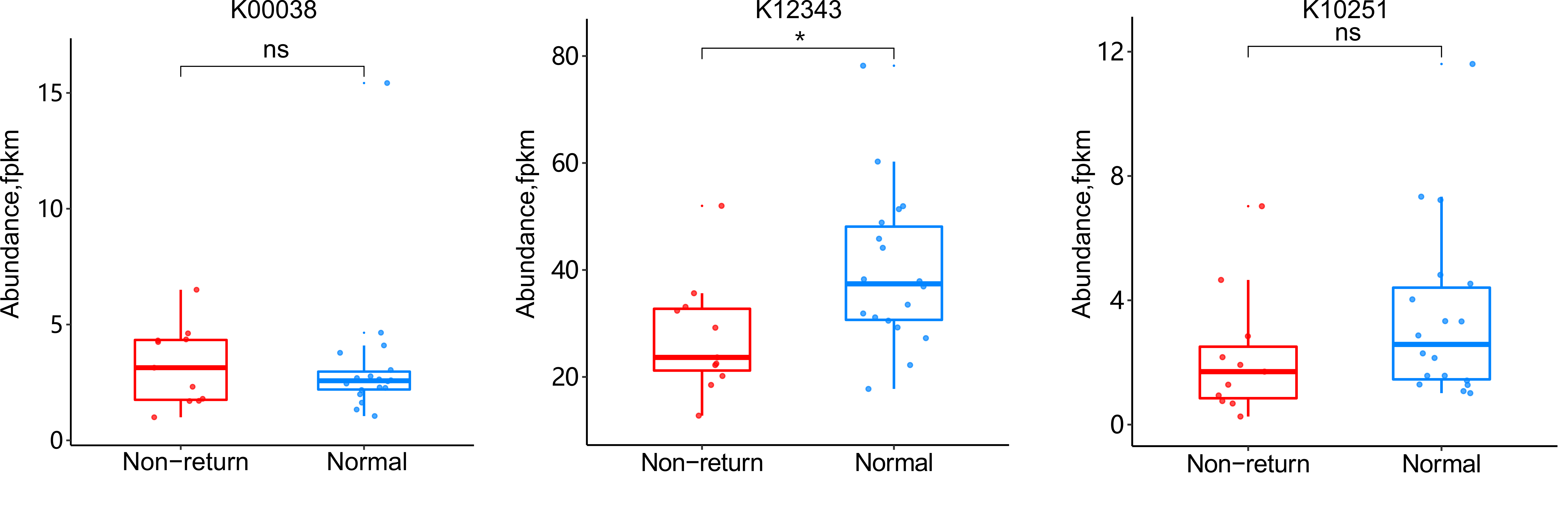


**Fig. S11** Comparing the abundances of microbial genes involved in the biosynthesis and metabolism of sex steroid hormones between normal return and non-return sows in the validation cohort


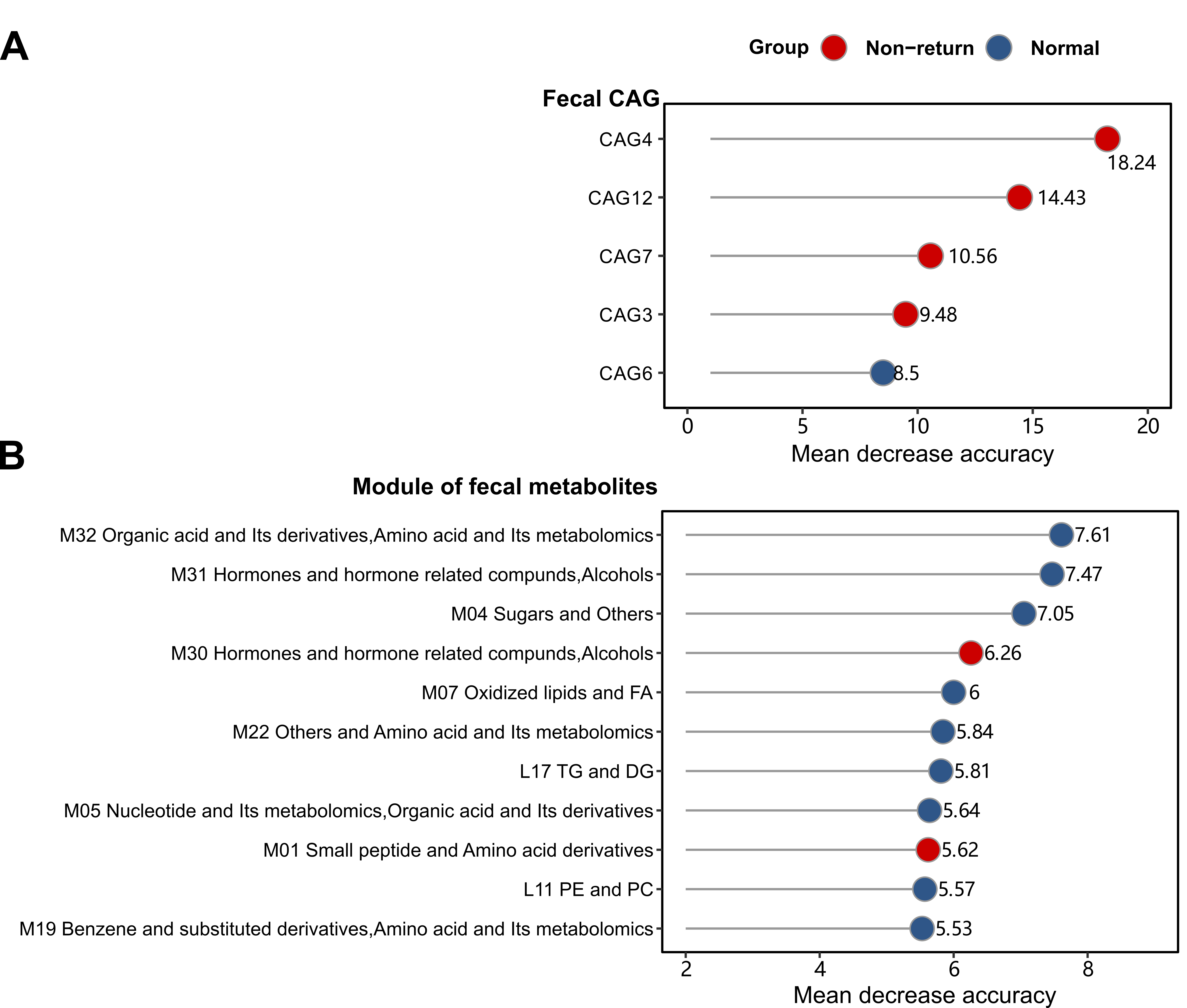


**Fig. S12** Biomarkers based on gut microbial CAGs and metabolite modules for discriminating non-return from normal return sows. **A** Biomarkers of gut microbiota CAGs. **B** Biomarkers of fecal metabolite modules. The length of the lines indicates the importance of biomarkers. The red dots represent the biomarkers significantly enriched in non-return sows, while blue dots represent the biomarkers significantly enriched in normal sows
